# Derivation of nociceptive sensory neurons from hiPSCs with early patterning and temporally controlled *NEUROG2* overexpression

**DOI:** 10.1016/j.crmeth.2022.100341

**Published:** 2022-11-15

**Authors:** William Plumbly, Nikolaos Patikas, Sarah F. Field, Stefanie Foskolou, Emmanouil Metzakopian

**Affiliations:** 1 UK Dementia Research Institute, Department of Clinical Neurosciences, University of Cambridge, Cambridge CB2 0AH, UK; 2 Open Targets, Wellcome Genome Campus, Hinxton, Cambridge CB10 1SA, UK

**Keywords:** chronic pain models, human iPSC, iPSC differentiation, sensory neurons, multi-electrode array platform, electrophysiology, single-cell RNA-seq

## Abstract

Despite development of protocols to differentiate human pluripotent stem cells (hPSCs), those used to produce sensory neurons remain difficult to replicate and result in heterogenous populations. There is a growing clinical burden of chronic pain conditions, highlighting the need for relevant human cellular models. This study presents a hybrid differentiation method to produce nociceptive sensory neurons from hPSCs. Lines harboring an inducible *NEUROG2* construct were patterned toward precursors with small molecules followed by *NEUROG2* overexpression. Neurons expressed key markers, including *BRN3A* and *ISL1*, with single-cell RNA sequencing, revealing populations of nociceptors expressing *SCN9A* and *TRP* channels. Physiological profiling with multi-electrode arrays revealed that neurons responded to noxious stimuli, including capsaicin. Finally, we modeled pain-like states to identify genes and pathways involved in pain transduction. This study presents an optimized method to efficiently produce nociceptive sensory neurons and provides a tool to aid development of chronic pain research.

## Highlights

• A hybrid differentiation protocol for deriving nociceptive sensory neurons from iPSCs
• Cells are characterized in depth with scRNA-seq and multi-electrode array physiology
• We model pain-like states to investigate pathways involved in pain transduction

## Motivation

Having an accurate and reliable human cell model of nociceptive sensory neuron function is invaluable for developing our understanding of the pathophysiology of chronic pain disorders, which are a rapidly increasing burden on societies worldwide. Current human iPSC differentiation approaches can produce heterogenous cultures of cells, often with small populations of nociceptors. We present an optimized differentiation approach to reliably produce populations of nociceptors that can be utilized as a model to investigate human pain pathways.

## Graphical abstract

**Figure:**
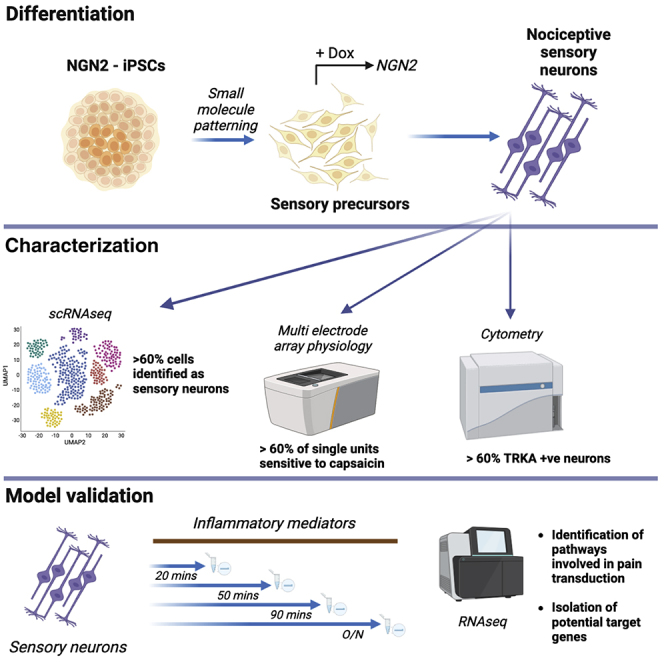

## Teaser

Plumbly et al. present a differentiation protocol for nociceptive sensory neurons, combining small-molecule patterning and overexpression of *NEUROG2*. Cultures are characterized with single-cell RNA-seq and multi-electrode array electrophysiology and then utilized to model pain-like states to investigate gene pathways involved in pain transduction.

## Introduction

An essential tool for understanding disease mechanisms in neurological disorders is development of physiologically relevant disease models. The advent of human induced pluripotent stem cells (iPSCs) and subsequent differentiation protocols developed over the last 10 years has allowed unprecedented insights into disease mechanisms in a human cell context. Much of this work has been focused on neurons of the central nervous systems, but relatively few protocols exist for differentiation of peripheral nervous system cells, including nociceptive sensory neurons. Chronic pain disorders (CPDs) are a significant and rapidly increasing cause of major disability worldwide, with around 30% of the global population experiencing chronic pain at some point throughout their lives.^1^ CPDs are highly heterogeneous and currently available treatments have variable efficacy and limited utility. Therefore, an accurate and reliable human model of nociceptive sensory neuron function is invaluable for our understanding of the pathophysiology of CPDs.

*In vivo*, sensory neurons are derived from migratory neural crest cells, where tightly regulated molecular processes lead to formation of several subtypes. A key part of this pathway is differential expression of the neurotrophin Trk receptors, where selective expression of TrkA, TrkB, and TrkC leads to formation of neurons that develop into nociceptors, mechanoreceptors, and proprioceptors, respectively.^2^ This differential expression is regulated by two members of the neurogenin family of basic-helix-loop-helix (bHLH) transcription factors: neurogenin 2 (*NEUROG2*) and neurogenin 1 (*NEUROG1*), which drive waves of TrkB/C^+^ and TrkA^+^ neuron development, respectively.^3^ Further diversity is established by functional expression of specific voltage- and ligand-gated ion channels. For example, expression of transient receptor potential (TRP) channels confers sensitivity to heat (TRPV1), cold (TRPM8), and noxious compounds (TRPA1).^4^^,^^5^^,^^6^ Therefore, protocols for developing human pain models from iPSCs have focused on these processes to produce populations of TrkA^+^ nociceptive sensory neurons.

Currently, two approaches exist for differentiation of hiPSCs into sensory neurons. A small-molecule approach, based on initial dual-SMAD inhibition and followed by inhibition of NOTCH signaling, GSK-3, and vascular endothelial growth factor (VEGF)/fibroblast growth factor (FGF) receptor tyrosine kinase,^7^^,^^8^^,^^9^ drives cells toward a sensory neuron fate, typically defined by expression of *BRN3A* and *ISL1*. Maturation of neurons is achieved by addition of growth factors, including Neurotrophin-3 (NT3) and beta nerve growth factor (bNGF), acting on TrkB/C and TrKA, respectively. A second approach involves forced expression of *NEUROG1* or *NEUROG2* together with the transcription factor *BRN3A.* Overexpression of either neurogenin together with *BRN3A* in human fibroblasts is sufficient to drive formation of functional sensory neurons, indicating that TrkA^+^ nociceptors can be produced via an *NEUROG2* pathway.^10^

Small-molecule approaches have demonstrated production of sensory neurons that compare favorably with primary dorsal root ganglion (DRG) tissue and exhibit a range of sensory neuron electrophysiological functions.^9^ However, it has also been shown that these populations remain heterogenous and exhibit significant culture- and cell line-specific variations.^8^ Although neurons produced with the forced transcription factor expression workflow express markers of sensory neuron development, a majority of cells do not functionally respond to prototypical nociceptor compounds, suggesting that many neurons are not functional pain receptors.^10^

In this study, we combine the two approaches outlined above to pattern hiPSCs toward a sensory neuron fate using small molecules, followed by a period of forced expression of *NEUROG2* to synchronize cultures and drive neuronal maturity. We find that this approach produces a homogenous population of sensory neurons, with single-cell RNA sequencing (scRNA-seq) providing identification of a clear cohort of nociceptors and high-throughput physiological characterization highlighting the presence of functional pain-related receptors. Finally, we use our sensory neurons to model pain-like states and identify genes and pathways that could provide novel targets for treatment of chronic pain.

## Results

### Patterning of human iPSCs to sensory neuron precursors

Our initial experiments focused on recapitulating the protocols first described by Chambers et al.^8^ and optimized by Schwartzentruber et al.^9^ We found that, although neurons produced using these protocols (across several iPSC lines) exhibited some markers of sensory neurons (e.g. *BRN3A* and *ISL1*), other expected genes were not observed, and a high degree of heterogeneity was seen in the cultures. Some cultures contained populations of proliferative non-neuronal cells that, despite use of mitotic inhibitors, remained present. Figure S1A summarizes the expression of marker genes across a panel of cell lines and highlights the variation in expression between lines. This panel included two cell lines harboring tetracycline-inducible *NEUROG2* constructs (Figure S1A; G3-NGN2 and B1-NGN2). Because of the role of *NEUROG2* in development of sensory ganglia *in vivo* and its demonstrated ability to drive sensory neuron differentiation in iPSC models,^10^ we decided to utilize the inducible construct as part of a combined protocol to determine its viability as an optimized differentiation approach.

**Figure 1:**
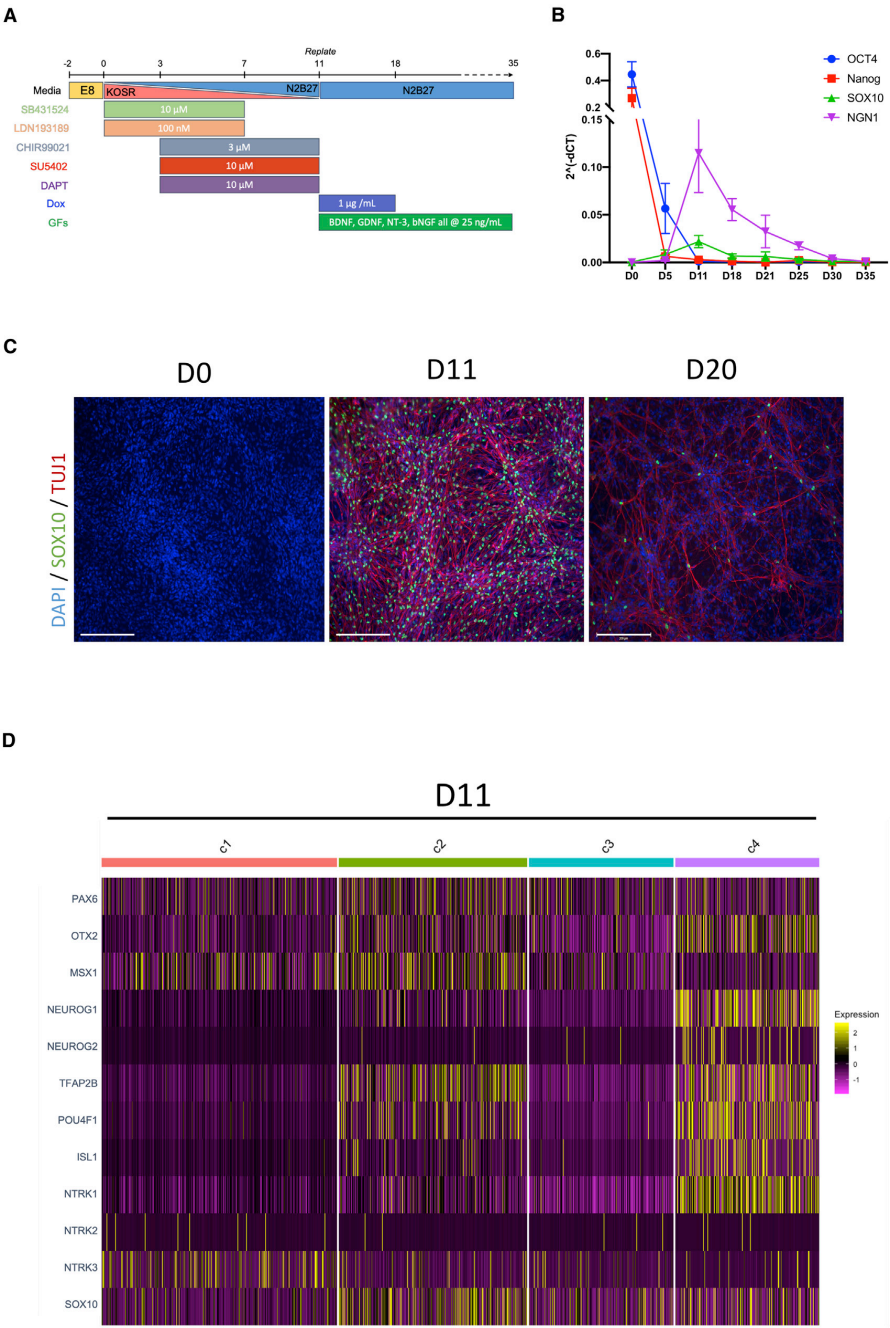
Small-molecule directed generation of sensory neural precursors from human iPSCs (A) Schematic of the optimized differentiation protocol used in the study. (B) Absolute values of early developmental gene expression normalized to housekeeping genes, as determined by qRT-PCR. Data shows means ± SD from 3 differentiations. (C) Representative ICC images highlighting the peak of *SOX10* expression on day 11. Scale bars show 200 μm. (D) Heatmap highlighting the normalized expression of key sNPC genes across 4 clusters identified from analyses of single-cell RNA sequencing data of day 11 precursors. Expression data represent normalized and scaled counts.

iPSCs harboring an inducible *NEUROG2* construct were first differentiated into sensory neuron precursors (sNPCs) with small molecules (Figure 1A). This 11-day patterning was applied during a graded change from a serum replacement medium to an N2/B27 based medium to promote neuronal development. Transcript time course analysis of cultures with qRT-PCR showed that, after a rapid decrease of pluripotent markers (*OCT4* and *NANOG*), expression of *SOX10*, a key transcription factor marker of mitotic neural crest cells, gradually increased to a peak on day 11 (Figure 1B). This was combined with an increase in expression of *NEUROG1*, a regulatory gene involved in production of TrKA^+^ sensory neurons. The pattern of progenitor formation was also demonstrated with immunocytochemistry (ICC), showing positive staining of *SOX10* in nuclei. This suggests that this patterning leads to formation of populations of sNPCs. To characterize the population of cells at this precursor stage, samples at day 11 were prepared for scRNA-seq. Analysis of these data showed that the population at this time point was relatively homogeneous, as shown by UMAP dimension reduction of the principal components (Figure S1B). Unsupervised clustering of the cells identified 4 “clusters” within the cohort, potentially representing small differences in stage of development. This was supported with pseudo-time analyses based on ordering cells by the first principal component, which showed a developmental movement from cluster 1 (c1) and c3 to c2 and finally c4, representing the most established of the cells at this time point (Figure S1C). These findings were also substantiated by looking at expression of sensory neuron developmental genes across the population. Although cell clusters representing an earlier developmental stage (c1 and c3) showed expression of formative regulatory transcription factors such as *PAX6*, *MSX1*, and *VIM*, cells in c2 and c4 expressed markers of sNPCs, including *TFAP2B*, *BRN3A* (*POU4F1*), and *ISL1* (Figure 1D). Cells in c4 represent a population more akin to early nociceptive sensory neurons, highlighted by strong expression of *NEUROG1* and *NTRK1*, the gene that codes for the TrkA receptor. These results provide strong evidence that the 11-day patterning produces a homogeneous population of sNPCs, a subpopulation of which appears to be committed to a nociceptor fate.

### NEUROG2 overexpression induces sensory neuron maturation

**Figure 2:**
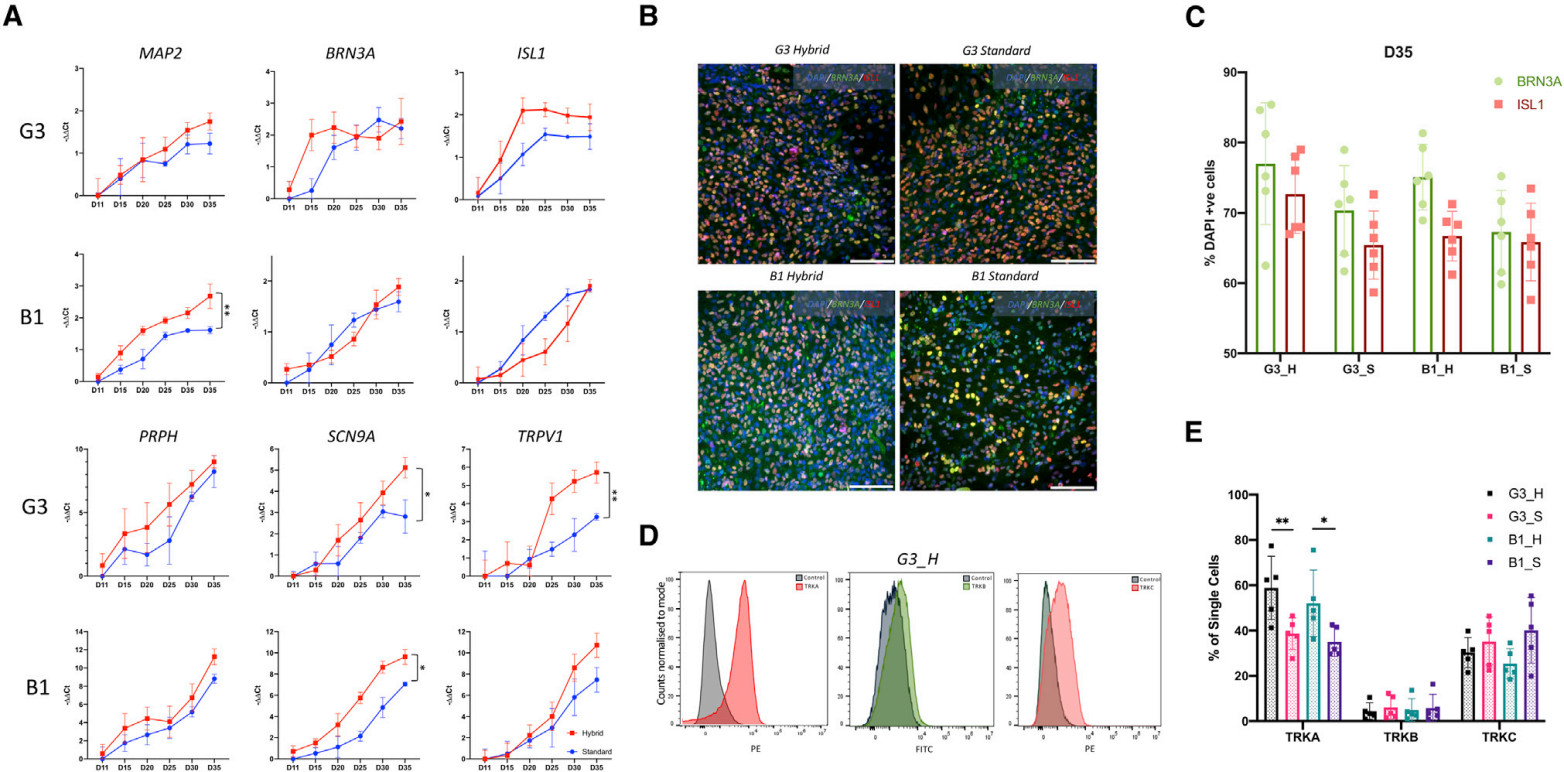
Comparison of SN differentiation protocols with combined small-molecule patterning and overexpression of *NGN2* (A) Gene expression profiles of small-molecule-only differentiations (standard) and hybrid differentiations (hybrid) were compared using qRT-PCR. In all panels, data show means ± SD normalized to housekeeping genes. Values for hybrid cultures were normalized to day 11 of standard culture values. ∗adjusted p < 0.05, ∗∗adjusted p < 0.01 as determined by Sidak’s multiple comparisons tests after two-way ANOVA. (B) Representative ICC images highlighting the expression of *BRN3A* and *ISL1* in cultures of all lines and conditions, performed on day 35. Scale bars show 100 μm. (C) Quantification of immunohistochemistry images. Data shows means ± SD. Dissociated neurons were stained with fluorophore-conjugated antibodies for TRKA, TRKB, and TRKC. (D) Representative histograms of cytometry data for the G3_H condition, showing counts of cell fluorescence for each of the antibodies, normalized to the mode of each distribution. (E) Cytometry data were quantified to provide an estimate of the number of single cells expressing each of the TRK proteins. Data shows means ± SD. ∗adjusted p < 0.05, ∗∗adjusted p < 0.01 as determined by Sidak’s multiple comparisons tests after two-way ANOVA. Data in (A), (C), and (E) comprise results from at least 3 differentiations for each condition.

Precursor cells were replated on D11. For our hybrid protocol (referred to here as “hybrid” and consisting of G3_H and B1_H conditions), cells were exposed to doxycycline to induce *NEUROG2* expression, whereas for the original protocol (referred to here as “standard” and consisting of G3_S and B1_S conditions), replated cells were treated on day 14 with a single acute treatment of the mitotic inhibitor mitomycin C (Figure 1A), as described by Schwartzentruber et al.^8^ Time course analysis with qRT-PCR showed that there was no difference in expression of the sensory neuron markers *BRN3A* and *ISL1* between standard and hybrid protocols in both cell lines, a trend also seen with the peripheral cytoskeletal gene *PRPH* (Figure 2A). However, across both cell lines, we observed an increase in expression of two nociceptor specific markers, *SCN9A* and *TRPV1,* in hybrid protocol cultures. 2-way ANOVAs revealed that, on day 35, these differences were significant, except for *TRPV1* in B1 cells, where only the trend was observed. qRT-PCR plots highlighting the comparable change in expression throughout development of the G3-hybrid line can be seen in Figure S2. Although we observed clear transcription-level expression of *SCN9A*, we detected only very low expression of *SCN11A* and could not detect *SCN10A* using these methods (Figure S2B). ICC of cultures on day 35 revealed protein-level expression of *BRN3A* and *ISL1* in all cell lines and conditions (Figure 2B). Quantification of stained cells confirmed that there were no differences in the number of BRN3A^+^ or ISL1^+^ cells between the standard and hybrid protocols of the G3 and B1 lines (Figure 2C). Together with the transcript-level analyses, this suggests that use of *NEUROG2* overexpression does not affect the number of BRN3A/ISL1^+^ cells in cultures. To determine whether the hybrid approach may affect the type of sensory neurons produced, we performed flow cytometry using conjugated antibodies for TRKA, TRKB, and TRKC (Figures 2D and S2D). Quantification revealed that, although there were no differences observed between the number of TRKB^+^ or TRKC^+^ cells, we recorded significantly higher numbers of TRKA^+^ cells in hybrid cultures of both cell lines (Figure 2E; both p < 0.001 as determined by Tukey’s tests after 2 way ANOVA). This suggests that our hybrid protocol is promoting development of TRKA^+^ sensory neurons (putative nociceptors) over those expressing TRKB or TRKC.

### scRNA-seq analysis of mature neurons demonstrates nociceptive sensory neuron identity

**Figure 3:**
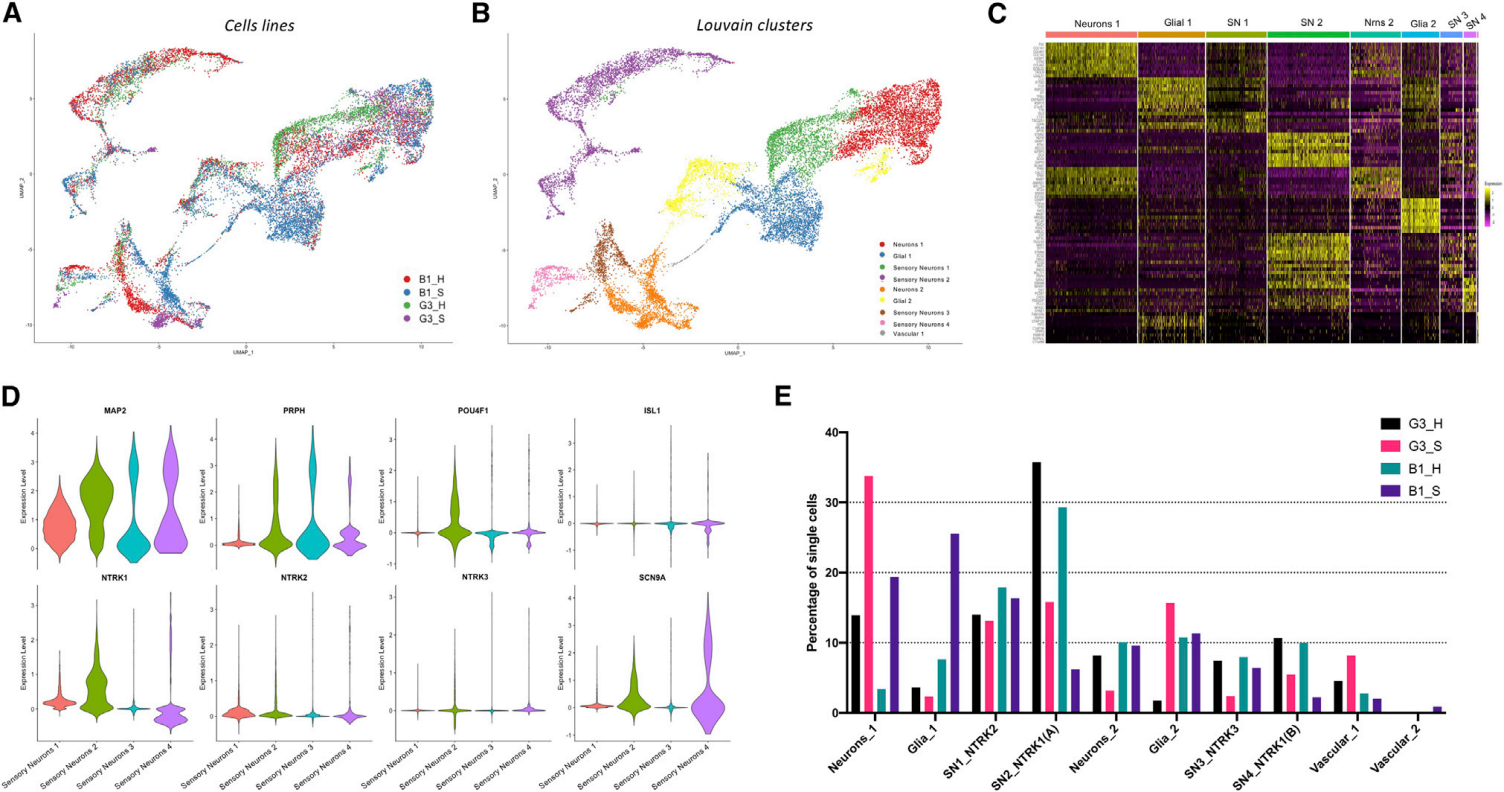
scRNA-seq characterization of iPSC-derived SNs Cultures were processed for scRNA-seq on day 35. Sample data were analyzed separately before being integrated to create a normalized and controlled dataset. (A and B) UMAP dimension reduction plots of the combined dataset, created from principal-component analysis (PCA) of normalized gene expression. Cells were grouped by the original source sample from which they derived. (B) shows cells grouped by identified clusters, as determined by shared nearest neighbor-based clustering. A total of 10 clusters were identified. (C) An expression heatmap highlighting the normalized expression of the top 10 marker genes identified for each of the 10 clusters. Clusters were assigned a cell type identity based on the differential expression of these markers. (D) Violin plots presenting normalized gene expression of key genes for the cells from all conditions assigned in one of the SN clusters. The proportion of each of the total single cells for each of the conditions was determined based on their assignment to each of the 10 clusters. (E) The percentage of each cell line that was clustered as part of each cell type.

To determine a more detailed characterization of the cell populations at the final time point, neurons from each of the four conditions were processed for scRNA-seq (see summary statistics of libraries in Figure S3A). Initial analyses of these data were performed on individual datasets before integration using an anchor-based approach to allow an accurate comparison across the four samples. First we compared the overall expression of the combined cell dataset in relation to each other cell. UMAP dimension reduction plots showed that there were identifiable clusters of cells, indicating a more heterogenous population than that observed on day 11 (Figure 3A). Cells from each of the four conditions were generally clustered together, indicating a degree of homogeneity across the different culture types. Next we ran an unsupervised clustering process to determine the presence of similar cell types based on cell-cell expression patterns. This approach identified 10 unique clusters in the combined dataset (Figure 3B). To further investigate the consistency of the differentiations across the conditions, the cluster assignments were grouped according to their original cell line and condition (Figure S3B). This highlighted that, although all four conditions were represented in each of the clusters, there were notable differences; for example, c1 appears overrepresented in B1_S cells, whereas the G3_S conditions have few cells in c5. The identity of the cluster assignments was achieved by utilizing Wilcoxon rank-sum tests to find genes characteristic of each population. Figure 3C shows normalized expression levels for each of the top 10 identified marker genes for each cluster across all cells and highlights the differences and similarities in expression patterns across the clusters. The identified marker genes were used to assign cell identities to each of the clusters, with the resulting cluster designations listed in Figure 3C. Cell types broadly fell into four groups: a general neuron identity (neurons 1 and 2), sensory neurons (SN) 1, 2, 3, and 4), glia-like cells (glia 1 and 2), and a vascular-like identity (vascular 1 and 2). Each of the SN subtypes were characterized by the predominant expression of each of *NTRK1*, *NTRK2*, or *NTRK3*. Expression plots for key SN marker genes are shown in Figure 3D for the four identified SN populations and highlight the differential expression of *NTRK* genes and the expression of *SCN9A*, which appears to be enriched in the *NTRK1*^*+*^ populations. To understand what proportion of mature cells from each of the four conditions were identified in each of the cell types, cells were quantified based on their cluster assignments (Figure 3E). This revealed notable differences in the types of cells produced by the standard and hybrid protocol. In particular, cultures from G3 and B1 hybrid conditions had a markedly higher percentage of SN2 and SN4 cells, expressing *NTRK1*, than their standard protocol counterparts. Conversely, standard protocol cultures contained higher proportions of cells identified as “general neurons” (Neurons_1 cluster; Figure 3E). We also observed that all conditions had relatively similar proportions of other SN clusters (SN1 and SN3), glia, and vascular-like cells, although there was a high proportion of Glial_1 cells present in B1_S. These results corroborate our findings from cytometry experiments, suggesting that our hybrid differentiation protocol induces development of a greater proportion of *NTRK1*^+^ SNs. When clusters were collapsed down to generic cell types, it was notable that the hybrid conditions produced higher proportions of SNs (driven by the higher number of *NTRK1*^+^ neurons), whereas greater numbers of generic neurons and glia were present under standard protocol cultures (Figure S3D).

Although the data presented above point to the viability of the differentiation approach in formation of nociceptive SNs, we wanted to analyze the cells in the context of other distinct but related populations. We first compared our scRNA-seq data from the hybrid differentiations with those generated from neurons produced using a cortical iNeuron protocol from iPSCs harboring an inducible *NEUROG2* construct. Data from two batches of G3_H SNs were compared with day 21 cortical iNeurons differentiated from the same line. UMAP dimension reduction highlighted the transcriptional differences between SNs and iNeurons, with a clear separation between cells of each fate (Figure S4A). The analysis also showed strong overlap between the two batches of SN populations, highlighting the reproducibility of our differentiation approach. Clear differences were also observed in expression of key SN gene markers between the sensory and cortical populations, highlighting the strong transcriptional differences between the cells of each differentiation type (Figure S4B).

We next wanted to evaluate our SN populations in the context of *in vivo*-derived DRGs. To our knowledge, there is no publicly available scRNA-seq dataset derived from human DRG. In lieu of such, we compared our data with a primate primary DRG scRNA-seq dataset produced using the SMRTseq2 platform.^11^ Datasets were first processed individually before combining them using an anchor-based integration approach. This provided a robust method of comparing like-for-like gene expression patterns while attempting to control for differences in the production of the datasets and species differences between the samples. The primate dataset consisted of DRGs from five *Macaca*, whereas the iPSC-derived dataset comprised cells from the G3 hybrid condition. First, UMAP dimension reductions revealed considerable overlap between our SNs and the primate DRGs, although some sample-specific regions were apparent (Figure S4C). Unsupervised clustering of the data, followed by differential gene expression to identify cell types, revealed that there were 10 unique cell types, including 5 DRG/SN clusters (Figure S4D). Parsing these cluster assignments by cell origin sample highlighted that, for many of the clusters, component cells derived from our iPSC-derived neurons and the primate DRGs, indicating a good amount of similarity between the cells. It was also notable, however, that our iPSC-derived cells contained populations of cells not present in the primary dataset (e.g., the yellow “ependymal” cluster and the pink generic “neurons” cluster), indicating differences in the populations (Figure S4D). Comparing the expression levels of several key genes demonstrated that our SNs compared favorably with the primate DRGs in terms of *BRN3A*, *ISL1*, and *SCN9A* expression but exhibited low expression of *SCN10A* and *SCN11A* (Figure S4E).

We then compared our data with a high-quality mouse primary DRG scRNA-seq dataset produced using the 10× Genomics platform.^12^ Despite the increased species differences with this dataset, we chose to perform this analysis because it allowed a direct comparison with a dataset produced using the same technologies as our data. The mouse DRG dataset was analyzed using the same methods as our data, and annotations of identified population clusters were performed as before by analyzing differentially expressed genes for each. The mouse DRG dataset was then integrated with an *NTRK1*^+^ subpopulation of our G3 SNs. We observed no overlap between our iPSC-derived SNs and any of the mouse DRG cells, although UMAP dimension reduction showed that our cells associated most closely with sub-populations of mouse DRG neurons and, importantly, were dissociated from the large population of Schwann cells and satellite glia present in the mouse dataset (Figure S4F). Comparing the expression levels of key genes demonstrated that, similar to the primate dataset, our SNs compared favorably with the mouse DRG in terms of *BRN3A*, *ISL1*, *NTRK1*, and *SCN9A* but exhibited relatively low expression of *SCN10A* and *SCN11A* (Figure S4G). These two sets of analyses suggest that, at the single-cell level, expression of *SCN10A* and *SCN11A* is low in our cultures. However, at the bulk transcript level in hybrid G3 cells, we saw expression of all three sodium channels, with *SCN9A* expression around 10-fold higher than that of *SCN10* and *SCN11A* (Figure S4H).

### Physiological monitoring of SNs with multi-electrode arrays reveals responses to a range of noxious stimuli

**Figure 4:**
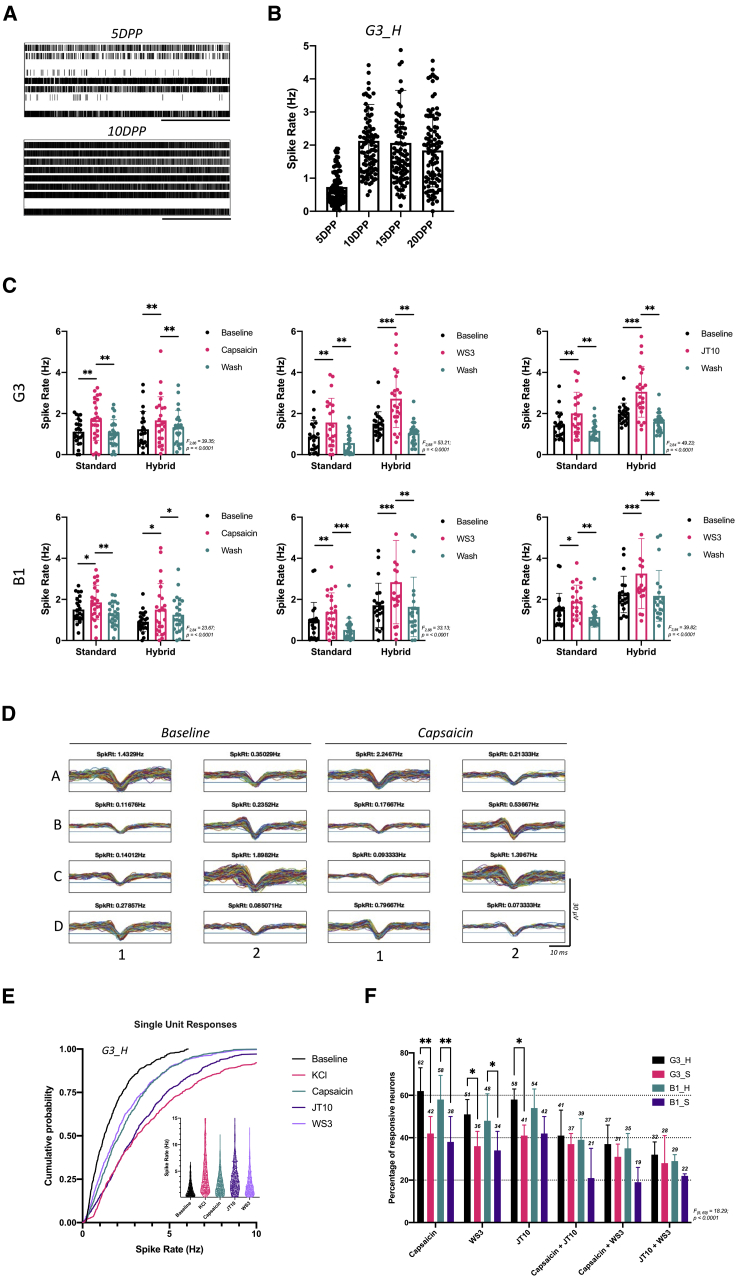
Electrophysiological and pharmacological profiling of SNs derived from human iPSCs Cells were replated on multi-electrode array (MEA) plates on day 11. (A) Raster plot of spontaneous activity of one G3_Hybrid culture (one well of a 96-well plate containing 8 electrodes). Each row represents the response from a single electrode, where each vertical line represents a spike (supra-threshold extracellular potential). The scale bar shows 100 ms. (B) The average spike rate of cells in each well (each dot represents one well). Plots show median spike rates for each time point, and error bars show SD. (C) Cultures were exposed to a panel of agonists to assess the presence of functional TRP channels. Plots present the average spike rate for individual wells before, during, and after exposure to noxious stimuli. Each dot represents the average spike rate for one culture; bars show means for each treatment group, and error bars show SD. ∗adjusted p < 0.05, ∗∗adjusted p < 0.01, ∗∗∗adjusted p < 0.001 as derived from Sidak’s multiple comparisons tests after 2-way repeated-measures ANOVA. (D) Spike shapes recorded for each electrode of a representative culture, where the average spike rate for each is shown above the trace. Horizontal lines in each show the spike threshold for each electrode. (E) The response of single units before and during treatments was analyzed (G3_H condition). The main plot shows the cumulative probability of firing rates across the conditions, where KCl represents a QC treatment to identify responsive cells. Inset: violin plots of the responses, where each dot shows the firing rate of a single neuron unit. (F) The percentage of single units that responded to each of the treatments. Data show means and error bars show SD. ∗adjusted p < 0.05 and ∗∗adjusted p < 0.01 as determined from Tukeys’s multiple comparisons tests after two-way ANOVA. Data are the result of 267 individual cultures across three independent differentiations.

Although the results described above point to a population of nociceptive SNs present in our cultures, it was important to establish whether these cells were functional and whether they responded to noxious stimuli. We therefore assessed the physiology of cultures by plating differentiating cultures onto multi-electrode arrays (MEAs), monitoring spontaneous and pharmacologically induced excitability and comparing responses across the four lines and conditions. Precursor cells were re-plated onto MEA plates on day 11 of differentiation. Activity was first measured 5 days post plating (DPP), when a low-level spontaneous excitability was observed in all cultures (for G3_H line, median = 0.6 Hz; Figures 4A and 4B). By 10 DPP, basal excitability had increased across the cultures and remained stable thereafter (Figures 4A and 4B), a trend observed across all lines and conditions. The spontaneous activity observed was in the form of single unit behavior; no coordinated network firing was observed in the SNsensory neuron cultures, in contrast to that seen in cultures of iPSCs differentiated to cortical neurons following an iNeuron protocol (Figures S5A and S5B).

We next assessed the response of SNs to a range of noxious stimuli, focused on those mediated by TRP channels. First, cultures were exposed to capsaicin to assess the presence of functional *TRPV1* channels. We saw that, for both differentiation conditions across both cell lines, capsaicin induced a significant increase in firing rate, which returned to baseline after drug washout (Figure 4C; all comparisons significant as determined by Sidak’s tests after 2-way ANOVA, see details in the figure). This strongly suggests that, at the culture-wide level, neurons from all conditions, regardless of protocol, contain SNs harboring functional *TRPV1* channels. We observed similar significant increases in firing rates when cells were exposed to agonists of *TRPM8* (WS3) and *TRPA1* (JT10) receptors, suggesting that neurons also possess these functional channels (Figure 4C). These results suggest that cultures of all conditions contain populations of functional SNs that, at a culture-wide level, cause a significant response to stimuli. We repeated these experiments on cultures of cortical iNeurons and found that there was no effect on the firing rate of these cells when exposed to these stimuli, indicating that the response was specific to the SN cultures (Figure S5B).

During the course of these experiments it became clear that the response of individual cells to these treatments varied considerably (Figure 4D). Therefore, to provide single-unit level analysis, spike shapes from each electrode of each well were captured and evaluated. Analysis of spike shapes revealed that, for most electrodes, the data represented spikes originating from a single neuron unit; for example, as shown in Figure 4D. However, where multiple spike shapes were observed in the same electrode trace (e.g., in Figure S5C), shapes were sorted using an unsupervised clustering method and parsed into their associated individual units (Figure S5C). To first quantify the total number of responsive neurons, cultures were exposed to high-KCl basal medium. Cells were then exposed to one of the TRP agonists, with positively responsive cells classed as a response to KCl and a stimuli, as defined in the STAR Methods. Single-unit responses to each of the three agonists were observed under all four culture conditions, confirming that all cultures contained populations of functional nociceptors (Figures 4E and S5D). However, cumulative probability plots highlighted a higher degree of shift in firing rates observed in response to agonists for the hybrid conditions compared with the standard protocol. To determine what proportion of cells responded to each stimulus type, the percentage of single cells that responded to each stimulus was calculated (Figure 4F). 62% of G3_H and 58% of B1 hybrid neurons responded to capsaicin, whereas the equivalent figures for standard-protocol neurons were significantly lower at 42% and 38%, respectively (both p < 0.01 as determined by Tukey’s tests after 2-way ANOVA). Similarly, a lower percentage of standard protocol neurons responded to the *TRPA1* agonist JT10 and the cooling agent WS3 compared with the hybrid conditions for each line. This strongly suggests that there are fewer functional cells present in the standard cultures. Although we observed a smaller percentage of neurons that responded to multiple stimuli, this was more consistent across the conditions (Figure 4F). These physiological results concur with our previous experiments, suggesting that cultures produced with our hybrid protocol contain a higher proportion of nociceptors than standard differentiations of the same cell lines.

### Investigating molecular pain pathways with *in vitro* models of extended pain states

We next utilized our hybrid protocol cultures to model pain-relevant contexts, first to investigate the pathways involved in pain transduction and to examine our cultures as a reliable model of SN function.

**Figure 5:**
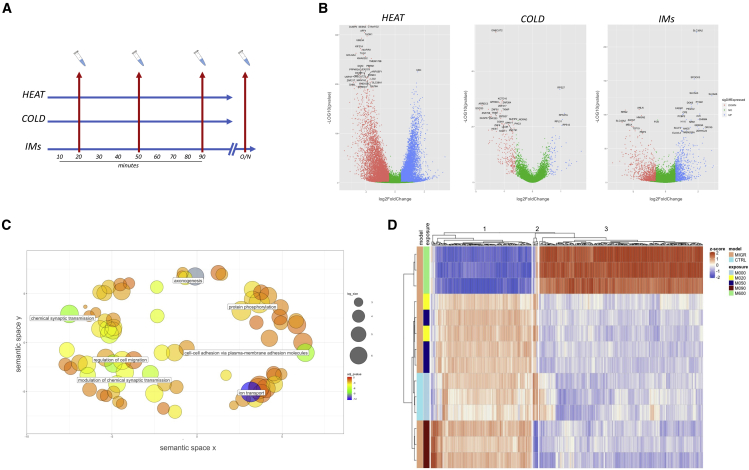
Investigating pathways associated with pain models using RNA-seq SNs were exposed to heat (45°C), cold (5°C), or an IM model over a total period of 18 h. (A and B) Control samples were taken before stimulus exposure. DEGs were found for all models, with the volcano plots in (B) showing the log2 fold change (x axis) and –log10(p value) (y axis) for each DEG. Red and blue dots represent genes with FDR < 0.05 and a log2 fold change of ±0.6 (classed as significantly differentially expressed). (C) The key enriched pathways for the inflammatory model as determined by analysis using Enrichr, followed by pathway condensation using Revigo. Each bubble shows an enriched pathway, positioned according to its functional relationship to other GO terms; labels denote representative pathways for each associated cluster. The size of bubbles shows the number of genes associated with each pathway term; the color scale shows the normalized adjusted p value of enrichment for each GO term. (D) Heatmap of DEGs determined after exposure to IMs, with samples clustered with regard to exposure time. Normalized gene expression changes for each gene/sample are shown as *Z* scores.

SN cultures from the G3 line were exposed to three pain-related stimuli. Extreme heat (45°C) and cold (5°C) treatments were chosen because they represent stimuli that act on a defined set of nociceptive pathways and could provide controls for stress-related gene changes. A cocktail of inflammatory mediators (IMs) was chosen, having been used previously as a model of migraine and described in *in vivo* and *in vitro* contexts.^13^^,^^14^^,^^15^ Samples were taken 20, 50, and 90 min after exposure, with a final overnight time point for the IM model (Figure 5A). Stranded RNA-seq was performed on each sample, with an average of 23.8 million reads mapped for each sample.

We found that all three of the models led to a number of significant differentially expressed genes (DEGs), defined as genes with a log_2_ fold change of ± 0.6 and passing a false discovery rate (FDR) value of less than 0.05 (Figure 5B; red and blue dots show DEGs according to this definition). The individual treatment models had different effects on the numbers and pattern of DEGs, with heat treatment inducing the highest number of significant genes (11,458 < 0.05 FDR) and those with the greatest changes in expression (mean = ±1.45 log_2_ fold change), whereas cold temperatures led to the lowest number of DEGs (2,545 < 0.05 FDR), with the smallest changes in gene expression (mean = ±0.49 log_2_ fold change).

To investigate the transcript expression changes observed over the exposure periods, heatmaps based on the exposure time point were made for each experimental condition (Figures 5 and S6). For the heat and cold conditions, expression changes broadly increased or decreased compared with controls over the exposure time course (Figures S6A and S6B). To confirm the existence of these trends, the temporal profile of DEGs was analyzed for pairwise similarity, with the results subsequently clustered. Plots of this analysis showed that, for the heat conditions, DEGs were increasing or decreasing over the exposure period (Figure S6C); for the cold condition, DEGs were up- or downregulated, with little effect from the exposure time (Figure S6D). Next we processed the significant DEGs through the Enrichr gene enrichment analysis platform. We found that, for both models, upregulated genes were significantly enriched in pathways involved in mitochondrial functions, including oxidative phosphorylation, mitochondrial transport, and cell stress signaling cascades (Figures S6E and S6F).

**Figure 6:**
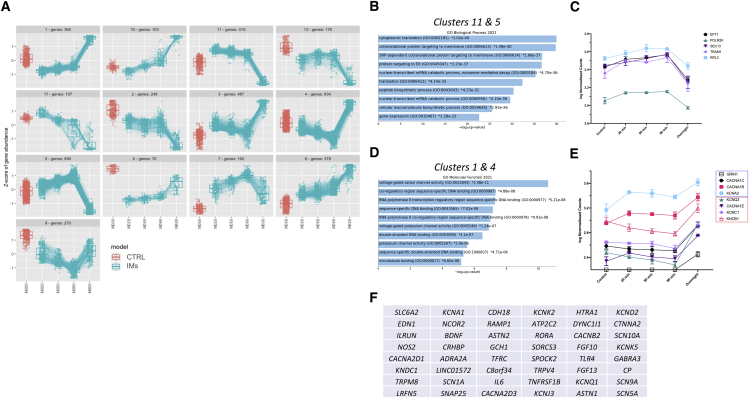
Gene expression changes in a cellular model of inflammation Significant differentially expressed genes (DEGs) were determined from RNA-seq experiments after exposure of SNs to a model of inflammation. (A) The result of clustering DEGs across the exposure period, based on a top-down divisive hierarchical algorithm. Normalized gene expression is shown as *Z* scores. The x axis labels represent the exposure time, where M000 = before exposure, M020 = 20 min, M050 = 50 min, M90 = 90 min, and M600 = 18 h. c11 and c5 were characterized by high or increasing early expression and a large decrease overnight, and c1 and c4 were characterized by a surge of expression between 90 min and overnight. (B and D) The results of gene enrichment analyses using the Enrichr platform, based on the genes in those clusters. Bar charts show the top 10 significantly enriched GO pathways, with the values showing the p value for each term. (C and E) Plots of the log of normalized counts for selected key genes. In (E), genes highlighted in the blue box are genes with known associations with chronic pain; those in the orange box are genes with no know pain associations. Data show means ± SD from 3 or 4 replicates. (F) Genes found in the HGPdb that were identified as significantly differentially expressed from the inflammatory model.

We also ran the significant DEGs from the IM model through the Enrichr platform. This showed that, for upregulated genes, enriched pathways included several involved in neuronal and synaptic function (Figure 5C). To observe patterns in gene expression changes, heatmap analysis was performed on the data as before (Figure 5D). As with the temperature models, this demonstrated that there were clear patterns of gene expression associated with different exposure time points; however, unlike those models, there were noticeable differences between the exposure time points, especially at the overnight time point. DEG patterns were again clustered (Figure 6A), which identified that there were complex patterns of expression associated with DEGs, with 13 total clusters identified. Several of the clusters were characterized by their expression level at the final overnight time point, being significantly higher or lower than the time point 18 h previously. Although some clusters appeared similar in their patterns of expression (e.g., c11 and c13), small differences in the initial exposure time point compared with controls led to grouping of such genes as a separate cluster. We focused on two gene sets based on two specific patterns of expression. The first showed an initial surge of expression, immediately or over the first 90 min, followed by a large decrease between the 90-min and overnight time points (c11 and c5 in Figure 6A). The 1,309 DEGs from these two clusters were processed through the Enrichr platform. Using the Gene Ontology (GO) term strand, we found that several of the significantly enriched pathways were those involved in global gene transcription and translation, including “cytoplasmic translation,” “protein targeting to ER,” and “nuclear-transcribed mRNA catabolic process” (Figure 6B). Although we did not identify any pathways that were descriptive of any specific transcriptional pathways, the convergence of these gene sets on a relatively narrow set of pathways relating to further protein production indicates that our cultures are engaged in an augmented gene expression response in the early stages of the IM model. It appears that, by 24 h after exposure, these genes are significantly downregulated, indicating that the cells have transitioned to a different phase of their response. Figure 6C shows the pattern of gene expression for five genes (*ETF1*, *POLR2K*, *HOMER1*, *TRAM1*, and *RPL3*) which were identified as part of c11 or c5 and were annotated as part of the significantly enriched pathways shown in Figure 6B. These genes code for proteins that are key parts of the transcriptional or translational machinery, with *RPL3* representative of over 50 ribosomal protein genes that were enriched in the pathways.

The second gene-set was characterized by an increase in expression seen between the 90-min and overnight exposures, as observed in c1 and c4 in Figure 6A. The 1,199 genes that form these two clusters were processed through the Enrichr platform utilizing the GO term strands. This revealed that several of the molecular pathways significantly enriched were those involved in regulation of specific gene transcription (“sequence specific double stranded DNA binding,” “*cis*-regulatory region DNA binding”) and cation channel activity (“voltage-gated cation activity,” “potassium channel activity”; Figure 6D). We found around 20 genes that code for subunits of the cation channels themselves, including voltage-gate calcium and potassium channels. Figure 6E shows the expression patterns of representative genes across the exposure time course, which were part of clusters 1 or 4 and the enriched pathways. Four of the genes identified (*GRIN1*, *CACN1C*, *CACNA1B*, and *KCNA2*, highlighted in the blue box) are those coding for cation channel subunits with known associations with chronic pain and have been identified as potential therapeutic targets. The remaining four genes shown in Figure 6E (*KCNQ3*, *CACNAE1*, *KCNC1*, and *KCNB1*, highlighted in the orange box) are those coding for similar proteins (subunits of voltage gated calcium and potassium channels) but have no known associations with chronic pain and, therefore, may be newly identified contributors to molecular transduction of pain states.

Finally, to gain an understanding of whether the gene changes observed with our SN pain model relate more generally to genes involved in pain-like states, we compared our cohort of significantly upregulated DEGs from the IM conditions with the Human Pain Genes Database (HPGdb), a curated database of genes harboring chronic pain-associated variants.^16^ We found that, of the 1,042 genes defined as significant DEG, 49 of these were found in the HPGdb, around 10% of the total database (Figure 6F). Many of these genes are directly involved in neuronal signaling, including voltage-gated potassium channels, calcium channels, and sodium channels, as well as other genes associated with SN function (e.g., *NOS2* and *BDNF*).

## Discussion

We find here that, by combining small-molecule patterning and temporally controlled overexpression of *NEUROG2*, we could differentiate hiPSCs toward a SN fate. Across two different cell lines, we found our hybrid approach to be more efficient and produce more homogenous cultures than a small-molecule-only protocol. Functional studies demonstrated that cultures responded positively to noxious stimuli targeting TRP channels, highlighting that a large population of neurons has functional nociceptor-associated channels. Together with the sequencing data, this presents clear evidence that our differentiation approach leads to a higher yield of nociceptive SNs than previous approaches. We trialed our cultures as models of SN function by exposing cells to pain-related contexts, demonstrating that our optimized protocol produces cultures that can be used as reliable *in vitro* models of human nociceptive function and as a platform for investigating molecular pathways involved in transduction of pain states.

Small-molecule approaches to SN differentiation yield neurons with characteristics similar to those observed in primary DRGs, including gene expression, responses to noxious stimuli,^7^ and functional expression of ion channels and receptors.^9^ However, these protocols have also been shown to lead to variations in final neuronal populations, including the presence of proliferative non-neuronal cells.^8^ We used an optimized small-molecule protocol as our “standard” approach to provide a comparison for our hybrid protocol. Studies utilizing direct conversion of iPSCs via overexpression of *BRN3A* and *NEUROG1*/*NEUROG2* also showed expression of key sensory markers and cells were shown to be functionally active, although many neurons did not respond to noxious stimuli.^10^ Our protocol combines these two approaches and leads to what we believe is a more robust and functionally responsive population of nociceptive SNs. scRNA-seq experiments identified that around 60% of hybrid cultures were SNs, and of these, around 30% were confidently identified as nociceptors, whereas cytometry experiments showed that around 55%–60% of semi-mature cells expressed *TRKA*. This is compared with around 40% of standard protocol cells being identified as SNs by scRNA-seq, with cytometry data showing around 40% of standard protocol cells as TRKA^+^, strongly suggesting that the standard protocol produces fewer nociceptive cells. We also observed greater reliability in data from our hybrid cultures compared with the standard cells, with cell line variability more pronounced in small-molecule-only cultures.

*In vivo*, *NEUROG2* expression regulates development of *NTRK2*- and *NTRK3*-expressing (TrKB- and TrKC-expressing) mechanoreceptors and proprioceptors, respectively, whereas nociceptor specification is regulated by *NEUROG1.*^2^ However, it has been shown previously that there are no differences in the relative numbers of different classes of SNs with *NEUROG1*- or *NEUROG2*- forced induction of iPSCs,^10^ and our data strongly suggest that *NEUROG2* is sufficient to drive nociceptor development. Comparisons of cells produced with our protocol and from primate DRGs demonstrated that the two populations clustered together and had comparable expression of sensory genes (e.g., *BRN3A* and *ISL1*). For nociceptor markers (e.g., *SCN9A* and *SCN10A*), expression was noticeably lower in our cells compared with primary DRGs, which is likely due to the relative immaturity of iPSC-derived cells. It would be useful to compare our cells with an analogous human DRG scRNA-seq dataset to allow a more representative comparison. Because of the size of human DRGs, scRNA-seq of these cells is challenging. Recently, single-nucleus RNA-seq datasets have been published,^17^ and a spatial transcriptomics analysis of human DRGs has also been described.^18^ An interesting aspect of these studies is the differences that are being revealed between rodent and human DRG transcriptomes, especially regarding the expression of *NTRK* genes and *TRP* channels. We found in our study that most SNs expressed *NTRK1*, with some showing overlapping expression with *NTRK2* or *NTRK3*. Although this is thought not to occur in mice, it has been shown that some human nociceptors co-express *NTRK1* and *NTRK2* in populations of *TRM8*^+^ neurons, whereas *NTRK1* and *NTRK3* are co-expressed in *TRPV1*^+^ β-amyloid (Aβ) nociceptors. An extended analysis with these new datasets could provide further insights with regard to the differences between our SNs and human DRGs, although challenges in comparing single-nucleus and single-cell datasets should be noted.

A key marker in identification of nociceptive SNs is expression of *SCN9A*, which codes for the Na_v_1.7 sodium channel and is highly expressed in DRGs.^19^ However, the expression of two other channels, Na_v_1.8 and Na_v_1.9 (coded by *SCN10A* and *SCN11A*, respectively) may represent nociceptor-specific sodium channels.^20^^,^^21^ Studies involving iPSC-derived SNs have shown the presence of *SCN9A* and *SCN10A* (but not *SCN11A*) in cultures at the transcript level and in functional experiments.^7^^,^^9^^,^^10^^,^^22^ We found expression of *SCN9A* at the bulk level and expression in a population of neurons identified with scRNA-seq. We also observe low-level expression of *SCN10A* and *SCN11A* in bulk experiments but not at the single-cell level, suggesting that the expression level of these genes cells is below the limit of single-cell detection. Across all cell lines and conditions, we also observe small populations of cells that are identifiably not SNs, including two vascular populations (primarily endothelial-like cells) and two glial clusters. Most of these glial-like cells expressed genes, including *SOX10*, *S100B*, *APOD*, and *PLP1*, that represent markers for Schwann cells, the principal glia of the PNS.^23^ The presence of a small population of Schwann-like cells is perhaps welcome, increasing the physiological relevance of the cultures. It would be useful to understand the state of these Schwann-like cells, in particular investigating whether functional myelinating Schwann cells were part of cultures.

Our MEA experiments allowed us to provide a large-scale functional characterization of our cultures to a level that, to the best of our knowledge, has not been shown previously. Over 55% of neurons produced using our hybrid protocol responded to at least one stimulus, and we found that, for standard cultures of the same cell lines, the equivalent figure was around 40%. Control experiments with cortical neurons showed that there was no response of this cell type to the stimuli, strongly suggesting that our observations in sensory cultures are specific. Across three outcome measures analyzed (flow cytometry, scRNA-seq, and electrophysiology), we observed consistently higher numbers of identifiable nociceptors in hybrid cultures compared with standard cultures across both cell lines used. However, functional experiments suggested a larger population of nociceptors present in cultures than that identified by transcriptomics. It is possible that the expression level of several marker genes for nociceptors (including ion channels) is below the sensitivity level of the scRNA-seq platform, leading to fewer identifiable nociceptor SNs with this technique. We also found that between 30% and 40% of neurons responded to at least two stimuli, which was consistent across conditions regardless of protocol. *In vivo*, the expression pattern of TRP channels is complex. For example, TRPV1 is primarily expressed in c-fiber afferents and small/medium cells of mouse DRGs,^24^ and recent experiments have shown that expression of *TRPV1* in humans is more widespread.^18^ Co-expression of TRPV1 and other TRP channels can vary depending on the location of the ganglion in the periphery.^25^^,^^26^ This provides a mechanism by which neurons can be tuned to respond to a wide range of stimuli.

Our pain modeling experiments are, to the best of our knowledge, the first time that global gene expression changes have been investigated in a human cellular model of pain. We first exposed cells to extreme heat and cold temperatures, which elicited significant changes in expression of genes enriched in pathways involved in cell stress responses, including oxidative phosphorylation. The effect of temperature on oxidative phosphorylation is well described^27^^,^^28^^,^^29^ and includes uncoupling of oxidation and phosphorylation, leading to mitochondrial deterioration, increased reactive oxygen species, and augmented cell death.^30^ It is likely, therefore, that the gene expression changes observed with these temperatures represent transcriptional responses of our cells in a pathological state. Although it is possible that pain-related pathways were perturbed during the experiments, these were overwhelmingly masked by genes involved in cell stress and death mechanisms.

A complex pattern of gene expression changes was observed when our cultures were exposed to IMs. For significantly upregulated genes, highly enriched gene sets included those involved in neuronal processes, including synaptic transmission, action potential formation, and cation transport. Significant DEGs identified as part of these pathways included key nociceptor genes such as *SCN9A*, *ASIC1*, and *TRPA1*, suggesting that, even at the experiment-wide level, the IM model induces expression changes related to transduction of neuronal signals. We focused our analysis on two gene sets based on observed gene expression patterns across the experiment time course. The first cohort (characterized by increased initial expression followed by a large decrease by the final time point) contained genes that were enriched in pathways related to global gene expression, including more than 50 ribosomal protein genes. One of the genes identified was *HOMER1*, known for coding a post-synaptic density scaffolding protein. Several isoforms of *HOMER1* are present in neurons and act as regulators of synaptic plasticity via interactions with glutamate receptors.^31^^,^^32^ However, *HOMER1A* and *ANIA3* isoforms are transcribed in response to increased neuronal activity and act as immediately-early genes (IEGs), forming the earliest transcriptional response.^33^^,^^34^^,^^35^ Although identification of isoforms was not part of our study, the expression profile of *HOMER1* in response to IMs suggests that it could be IEG forms observed here, acting as part of an initial upregulation of pathways involved in global transcription and translation.

Genes from the second set (low initial expression followed by a rapid increase overnight) were enriched in pathways involved in synaptic function, including the activity of voltage-gated cation channels. These pathways were only upregulated after 18 h of exposure, potentially indicating that their perturbation was in response to the wave of transcription observed earlier. We identified that several of the upregulated genes code for subunits of the cation channels themselves, including some previously identified as contributors to pain pathways. *KCNA2*, coding for the K_v_1.2 potassium channel, is involved in establishment and maintenance of neuropathic pain states^36^^,^^37^ and has attracted renewed interest as a therapeutic target via the action of non-coding RNAs.^38^^,^^39^ Likewise, *CACNA1C* and *CACNA1B* are subunits of L-type calcium channels with known roles in acute and chronic pain processing.^40^^,^^41^ We also identified several cation channels that, to our knowledge, have no known role in processing of chronic pain. *KCNQ3* codes for the K_v_7.3 voltage-gated potassium channel and is part of the KCNQ/M family. Although there is no direct evidence of the role of *KCNQ3* in pain processing, this family of channels has been implicated in manifestation of chronic pain states,^42^^,^^43^^,^^44^ indicating that *KNCQ3* itself may have a currently undisclosed role. Further investigation regarding the role of these identified channels in pain transduction could lead to identification of novel treatment targets.

### Limitations of study

Although we are confident that the protocol described here robustly and reliably produces cultures of SNs, our validation of the protocol is currently limited to two iPSC lines. The reliability of the protocol to produce nociceptive SNs across a broad range of iPSCs is therefore unknown. Although we produced estimates of nociceptive SNs proportions using three different experimental approaches, discrepancies exist between the different methods, particularly between scRNA-seq and MEA experiments. We feel that these differences are likely due to the sensitivity limit of the 10× Genomics scRNA-seq approach we chose for this study, especially because we aimed to balanced sequencing depth and breadth. With increased sequencing depth, we could potentially capture a greater number of low-expressed mRNAs. Given the specificity of the agonists used in our physiology experiments, we regard the proportions of cell types identified with the MEA platform as a more reliable indicator.

Although we perform several steps of control to accurately determine the nature of a single-unit response in our MEA experiments, we cannot fully rule out the possibility of misidentifying single units. Although more than 90% of electrodes detect the activity of a single cell unit (as determined by spike shape), the remainder detect multiple shapes that are deconvoluted into single units using a spike-sorting algorithm. It is possible, therefore, that this miscaptures a unique spike shape as a single unit, instead of, for example, detecting activity in the cell body and axon of the same neuron. However, because we detect multiple events from the same electrode less than 10% of the time, and this is consistent regardless of cell line or condition, we do not feel that this affects our findings.

We also acknowledge that an assessment of cell function with single-cell electrophysiology (which we have not presented here) would add a deeper understanding of the functional state of the neurons, particularly regarding physiological maturity. These techniques would also allow an assessment of the contribution of the sodium channels *SCN9A*, *SCN10A*, and *SCN11A* in our cells, specifically regarding the presence of tetrodotoxin-resistant currents.

Finally, the extent to which our pain-modeling experiments can be considered chronic is perhaps limited, especially for the experiments lasting 90 min or less. Therefore, the degree to which our IM model can be further utilized to better represent a chronic state should be investigated.

## STAR★Methods

### Key resources table

**Table:** 

REAGENT or RESOURCE	SOURCE	IDENTIFIER
**Antibodies**		

SOX10	Cell Signaling	89356; RRID: AB_2792980
NEUROG1	R&D Systems	mab3524; RRID: AB_2149376
BRN3A	Millipore	mab1585; RRID: AB_94166
ISL1	Abcam	AB178400
Tuj1	Millipore	MAB1637; RRID: AB_2210524
PRPH	Abcam	AB4666; RRID: AB_449340
Alexa Fluor 488 -Donkey anti-rabbit IgG (H + L)	Thermo Fisher	Cat# A-21206; RRID:AB_2535792
Alexa Fluor 488 - goat anti-mouse IgG (H + L)	Thermo Fisher	Cat# A28175, RRID:AB_2536161
Alexa Fluor 594 -Donkey anti-rabbit IgG (H + L)	Thermo Fisher	Cat# A-21207; RRID:AB_141637
Alexa Fluor 594 - goat anti-mouse IgG (H + L)	Thermo Fisher	Cat# A-11032, RRID:AB_2534091

**Chemicals, peptides, and recombinant proteins**		

Stem Pro Accutase	Thermo Fisher	A1110501
B-27supplement w/o Vitamin A	Thermo Fisher	12587010
DMEM/F12 (1:1)	Thermo Fisher	10565018
Glutamax	Thermo Fisher	35050061
Vitronectin	Thermo Fisher	A14700
Geltrex	Thermo Fisher	A1413301
Knockout serum replacement	Thermo Fisher	10828028
MEM Non-Essential Amino Acids Solution	Thermo Fisher	11140035
2-Mercaptoethanol	Thermo Fisher	31350010
LDN-193189	Tocris Bioscience	6053
SB431542	Tocris Bioscience	1614
DAPT	Tocris Bioscience	2634
SU5402	Tocris Bioscience	3300
CHIR99021	Tocris Bioscience	4423
Neurobasal	Thermo Fisher	21103049
BDNF	Peprotech	450-02
GDNF	Peprotech	450-10
NT-3	Peprotech	450-03
bNGF	Peprotech	450-01
N2 supplement	Thermo Fisher	17502048
Rock-inhibitor (Y-27632)	Tocris Bioscience	1254
Truseq Stranded mRNA Library Preparation Kit	Illumina	20020594
TeSR-E8	StemCell Technologies	05990
RNeasy plus RNA Extraction kit	Qiagen	74134
EDTA	Thermo Fisher	15575020
dPBS	Sigma Aldrich	D8537

**Critical commercial assays**		

Deposited data		
Bulk RNA sequencing	This Paper	GSE188272
Single Cell RNAseq	This Paper	GSE187345
Experimental models: Cell lines		
Human; iPSC line; G3-NGN2	Michael Ward; Fernandopulle et al., 2018	
Human; iPSC line; B1-NGN2	Mark Kotter; Pawlowski et al., 2017	

**Software and algorithms**		

Prism 9.0	Graphpad	RRID:SCR_002798
R Project for Statistical Computing	R-Project.org	RRID:SCR_001905
Seurat	Sajita Lab	RRID:SCR_016341
Monocle3	Cole Trapnell Lab; Cao et al., Nature 2019	RRID:SCR_018685
Cell Ranger	10× Genomics	RRID:SCR_017344
Fiji	fiji.sc; Schindelin et al., 2012^45^	RRID:SCR_002285
Samtools	htslib.org; Danecek et al., GigaScience 2021	RRID:SCR_002105
FloJo	BD Life Sciences	RRID:SCR_002285

### Resource availability

#### Lead contact

Further information and requests for resources and datasets should be directed to and will be fulfilled by the lead contact, Emmanouil Metzakopian.

#### Material availability

This study did not generate new unique reagents.

### Experimental model and subject details

The G3 human iPSC line used in this study was a gift from Michael Ward (NIH, Bethesda, MD) and was generated as described in Fernandopulle et al. 2018. The cell line harbors a doxycycline inducible *NGN2* constructed located at the safe-harbour AAVS1 locus. The B1 human iPSC line was a gift from Mark Kotter (University of Cambridge, UK) and were generated as described in Pawlowski et al. 2017. This line contains the inducible promotor and *NGN2* gene in the AAVS1 locus, with the rtTA repressive element located at the ROSA26 locus. All iPSCs were maintained in TeSR-E8 medium (Stem Cell Technologies) and cultured on Vitronectin coated plates (Thermo Fisher Scientific). Cells were passaged as required with 0.5 mM EDTA (Thermo Fisher Scientific) when reaching 80% confluency.

### Method details

#### Sensory neuron differentiation

Differentiations of iPSCs into sensory neurons was achieved using a hybrid small molecule/forced induction approach. The small molecule protocol was adapted from that described in Schwartzentruber et al., 2018.^8^ Briefly, iPSCs were dissociated into single cells using Accutase (Thermo Fisher Scientific) and plated at 75,000 cells/cm2 in TeSR-E8 medium with rock inhibitor onto Geltrex (Thermo Fisher Scientific) coated plates. After 24 h (on D0), media was changed for a knockout serum replacement media (KSR) containing KO DMEM, KSR (20%), 1× Non-Essential Amino Acids (NEAA), 2-Mercaptoethanol (10 μM) and 1×.

GlutaMax (all Thermo Fisher Scientific), supplemented with LDN-193189 (100 nM, R&D Systems) and SB431542 (10 μM, R&D Systems; ‘2i’). On D3, KSR medium was supplemented with both 2i molecules plus DAPT (10 μM, R&D Systems), SU (10 μM, Merck) and CHIR99021 (3 μM, R&D Systems; ‘3i’). From D4, KSR medium was gradually changed to a N2B27 medium containing Neurobasal medium, N2 (1×), B27 (1×) GlutaMax (1×) and 2- Mercaptoethanol (10 μM; all Thermo Fisher Scientific). The KSR:N2B27 media ratios were as follows: D4 – 75%:25%; D6 – 50%:50%, D8 – 25%:75%, D9 – 100% N2B27. 2i supplements were removed from media on D7.

On D11, cells were dissociated into single cells and replated onto either geltrex coated plates or geltrex coated coverslips. Cells were plated at 50,000 cells/cm2 in N2B27 medium containing rock inhibitor, BDNF, GDNF, NT-3 and bNGF growth factors (all 20 ng/mL, all Peprotech), and doxycycline (1 ug/mL; R&D Systems). Media was changed after 24 h for fresh N2B27 containing the same factors minus rock inhibitor. Cultures were maintained in this medium until the end of experiments (typically until D35 – D40).

#### RNA extraction and quantitative real-time PCR

Total RNA was extracted from cultures with the QIAGEN RNeasy Mini Kit (Cat No./ID: 74106), according to the manufacturers’ instructions. mRNA was converted to cDNA using SuperScript IV VILO (Thermo Fisher Scientific) according to the manufacturers’ protocol. Quantitative real-time PCR was performed using the Luna Universal Probe Master Mix (NEB) and TaqMan RT-PCR assays (Thermo Fisher Scientific). Reactions were performed in 384 well plates on an Applied Biosystems QuantStudio 5 instrument. The following probes were used: POU5F1 (Hs00999632_g1), SOX2 (Hs01053049_s1), NANOG (Hs02387400_g1), SOX10.

(Hs00366918_m1), NEUROG1 (Hs01029249_s1), POU4F1 (Hs00366711_m1), ISL1

(Hs00158126_m1), MAP2 (Hs00258900_m1), NTRK1 (Hs01021011_m1), NTRK2 (Hs00178811_m1), NTRK3 (Hs00176797_m1), PRPH (Hs00196608_m1), SCN9A,

(Hs00161567_m1), GAPDH (Hs99999905_m1), ACTB (Hs99999903_m1), TUBB3

(Hs00801390_s1), VGLUT2 (Hs00220439_m1), CUX1 (Hs00738851_m1), NECAB1

(Hs00332733_m), TRPV1 (Hs00218912_m1), TRPM8 (Hs00375481_m1), RUNX1

(Hs00231079_m1), TRPA1 (Hs00175798_m1), SCN10A (Hs01045137_m1), SCN11A (Hs00204222_m1), ASIC1 (Hs00952802_m1). Two housekeeping control assay were used for every experiment: GAPDH (Hs99999905_m1) and S18 (Hs99999903_m1). All rt-qPCR data was analyzed using a comparative CT method and all data is presented as absolute values, corresponding to 2^−dCT^. A single housekeeping value was obtained from the geometric mean of the two housekeeping genes for each sample/time point.

#### Immunofluorescence staining

After differentiation into sensory neurons on glass coverslips, cells were washed with PBS and fixed in 4% PFA for 15 min. After washing and permeabilization with 0.2% Triton X-100 for 1 h, coverslips were blocked with 10% BSA for 1 h at RT before incubation with primary antibodies at 4°C overnight. The following primary antibodies were used: Sox10 (#89356, Cell Signalling) at 1:1000 dilution; NEUROG1 (#mab3524, R&D) at 1:500 dilution, BRN3A.

(mab1585, Milipore) at 1:200, ISL1 (AB178400, Abcam) at 1:100 dilution, Tuj1 (MAB1637, Millipore) at 1:500 dilution, PRPH (AB4666, Abcam) at 1:100. After washing, coverslips were exposed to the required Alexa Fluor secondary antibodies (either Thermo Fisher Scientific or Abcam) and were incubated for 1 h at room temperature in the dark. Cultures were counterstained with DAPI and mounted onto slides using Dako mounting medium (Agilent). Images were captured using an EVOS FL Auto2 imaging system (Thermo Fisher) and processed with Fiji.^45^

#### Flow cytometry

Cells were dissociated with accutase and checked for viability. Suspensions were then washed twice with Flow Cytometry Staining buffer (R&D Systems). Cells were stained in suspension with conjugated antibodies for TrKA(PE), TrKB(FITC) and TrKC(PE), for 1 h at room temperature. Suspensions were washed twice with Staining buffer and then processed. Cytometry was done using a CytoFLEX S (Beckman Coulter) and data was analysed with FlowJo (BD Life Sciences).

#### Sample preparation for scRNA-seq

Cells were dissociated with accutase, counted and checked for viability. Single cell cDNA synthesis, amplification and sequencing libraries were constructed for 10000 cells per sample using the 10× Genomics Chromium platform following the manufacturer’s instructions. Libraries were sequenced using an NovaSeq 6000 sequencer (Illumina). Raw sequencing data was demultiplexed, aligned, mapped and feature matrices were generated with Cell Ranger (10× Genomics). Analysis of scRNA-seq data was performed in R with Seurat (v3).^46^

#### Modelling pain states and bulk RNAseq

Cultures of D40 sensory neurons were exposed to three models of pain-like states: 1.

Exposure to high temperatures, cells were placed in a general purpose incubator set to 45°C, validated with a digital thermometer; 2. Exposure to low temperatures, cells were placed in a laboratory fridge, set and validated at 4°C; 3. Exposure to a model of inflammation, cells were exposed to a cocktail of inflammatory mediators consisting of 10 μM bradykinin, 1 μM histamine, 1 μM prostaglandin E_2_ and 500 nM serotonin.^13^^,^^14^^,^^47^ For all three models, differentiation medium was removed, cultures were washed twice with dPBS and maintained for the experiments in HBSS with 10 mM HEPES and glutamax. Control samples were taken 30 min after changing media for HBSS. Experimental samples for all three models were taken 20, 50 and 90 min post exposure; with an additional ‘overnight’ sample taken 18 h after the 90 min samples (Total exposure time = 19.5 h). Total RNA was extracted from samples with the QIAGEN RNeasy Mini Kit as above. Individual mRNA sequencing libraries were made using the Illumina TruSeq Stranded mRNA Library Preparation Kit and indexed using TruSeq RNA single indexes following the standard manufactures instructions. Individual libraries were quantified with a Qubit 4 fluorometer; normalised and combined into three pools for sequencing. The final pools were quantified with qPCR using the NEBNext Library Quant kit for Illumina. Pools were sequenced using the Illumina Nextseq 550 platform using High Output 75 cycle kits with Read 1 set as 30 cycles and read 2 set as 56 cycles. FastQ files of the sequenced libraries were mapped to the GRCh38 genome using the STAR v2.7.3 aligner.^48^ After mapping, the transcript fragments were counted with the featureCounts module from the subread package,^49^^,^^50^ using the GRCh38 version 99 annotations. Several biological samples were sequenced across individual sequencing runs. For those samples, transcript counts were aggregated by summing them on a gene level after counting. Curated raw counts were analysed for differential expression using DEseq2 in R. Each model was analysed separately as an individual experiment and statistical model. Significant differentially expressed genes were found using likelihood ratio testing based on exposure time-points. Genes were deemed significantly differential expressed if they passed an adjusted p value (FDR) of < 0.05 *and* had a log_2_ fold change of +/− 0.6. Gene set enrichment analyses was done by processing gene lists through the Enrichr platform,^51^^,^^52^ utilising the Gene Ontology (GO) Biological Process and Molecular Function strands.^53^^,^^54^ Where required, GO term lists were condensed and prioritised using Revigo,^55^ with results imported into R for visualisations.

#### Multi electro array recordings

All MEA recordings were performed on an Axion Maestro Pro platform using either 96 or 48 well CytoView Plates. Cells were plated onto MEAs at D11 using a drop-culture method: Array wells were firstly coated by applying 15 ul of diluted Geltrex directly over the electrode area of each well. Plates were then incubated at 37 for 1 h. During this time, precursor cells were dissociated with accutase and counted. Geltrex was removed from MEA wells and immediately, 50000 cells in 15 ul of media were plated directly on top of the electrode area of each well. These drop cultures were then incubated for 1 h at 37°C. After 1 h, 350 ul of media was carefully added to each well. Cells were plated in N2B27 medium supplemented as described above, with the addition of rock inhibitor for 24 h. Media was changed every 48 h. Sterile water was also added to the reservoir compartments of each MEA plate to limit media evaporation and maintain humidity. This was also changed every 48 h.

MEA plates were typically recorded for either 5 or 10 min in a 37°C and 5% CO_2_ atmosphere, automatically controlled via the Maestro Pro. Recordings were taken at a sample rate of 12.5 kHz/channel with a Butterworth band-pass filter (200 Hz–3000 Hz). Positive and negative deflecting spikes were detected using an adaptive threshold method set at 6× standard deviation of the noise floor.

Initial stocks of compounds were prepared in DMSO. Working concentrations were then prepared by diluting initial stocks in recording medium. After a baseline recording of recording medium only, plates were returned to a tissue culture hood and working stocks of the following compounds were diluted in medium and added to wells as required: (*E*)-Capsaisin (10 uM, #0462), WS3(10 uM, #2927), JT010 (500 nM, #6269; all Tocris). The final concentration of DMSO in cultures was not greater than 0.02%. Plates were incubated for 5 min at 37°C before recording. To wash, media contain drug was removed and wells were carefully washed 3 times with DPBS. Fresh media was then added to wells and plated were incubated for 5 min before the washout recording. For single unit experiments, exposure to the above agonists was preceded by exposure to 25 mM KCl to identify responsive neurons. MEA recording data was analysed using the Axion Software suite and custom Matlab scripts.

### Quantification and statistical analysis

All statistical analysis was performed using GraphPad Prism or R. Details of data and statistics for individual experiments are presented in the main text, figures and/or figure legends. Unless otherwise stated, all data plots show means +/− SD. For bulk RNAseq analysis, significant differentially expressed genes were defined as those which passed an FDR of <0.05 and had a log fold change of +/− 0.6. These genes were taken forward for use in gene pathway analyses.

## Data Availability

• Single-cell and bulk RNA sequencing data have been deposited at GEO (NCBI) and are publicly available as of the date of publication. Accession numbers for datasets are listed in the key resources table. • This paper does not report any original code. • Any additional information required to reanalyse the data reported in this paper is available from the lead contact upon request

## Supplemental information

Document S1. Figures S1–S6

Table S1. Significant DEGs identified from RNA-seq (upregulated), related to Figures 5 and S6

Table S2. Significant DEGs identified from RNA-seq (downregulated), related to Figures 5 and S6

Document S2. Article plus supplemental information
