# Supplementary material for: Derivation of nociceptive sensory neurons from hiPSCs with early patterning and temporally controlled NEUROG2 overexpression
Source: Cell Rep Methods. 2022 Nov 15;2(11):100341. doi: 10.1016/j.crmeth.2022.100341 (PMC9701618; doi:10.1016/j.crmeth.2022.100341)
Supplement: Document S1. Figures S1–S6 [file mmc1.pdf]

Cell Reports Methods, Volume *2*

Supplemental information

Derivation of nociceptive sensory neurons from hiPSCs with early patterning and temporally controlled *NEUROG2* overexpression

William Plumbly, Nikolaos Patikas, Sarah F. Field, Stefanie Foskolou, and Emmanouil Metzakopian

Figure S1

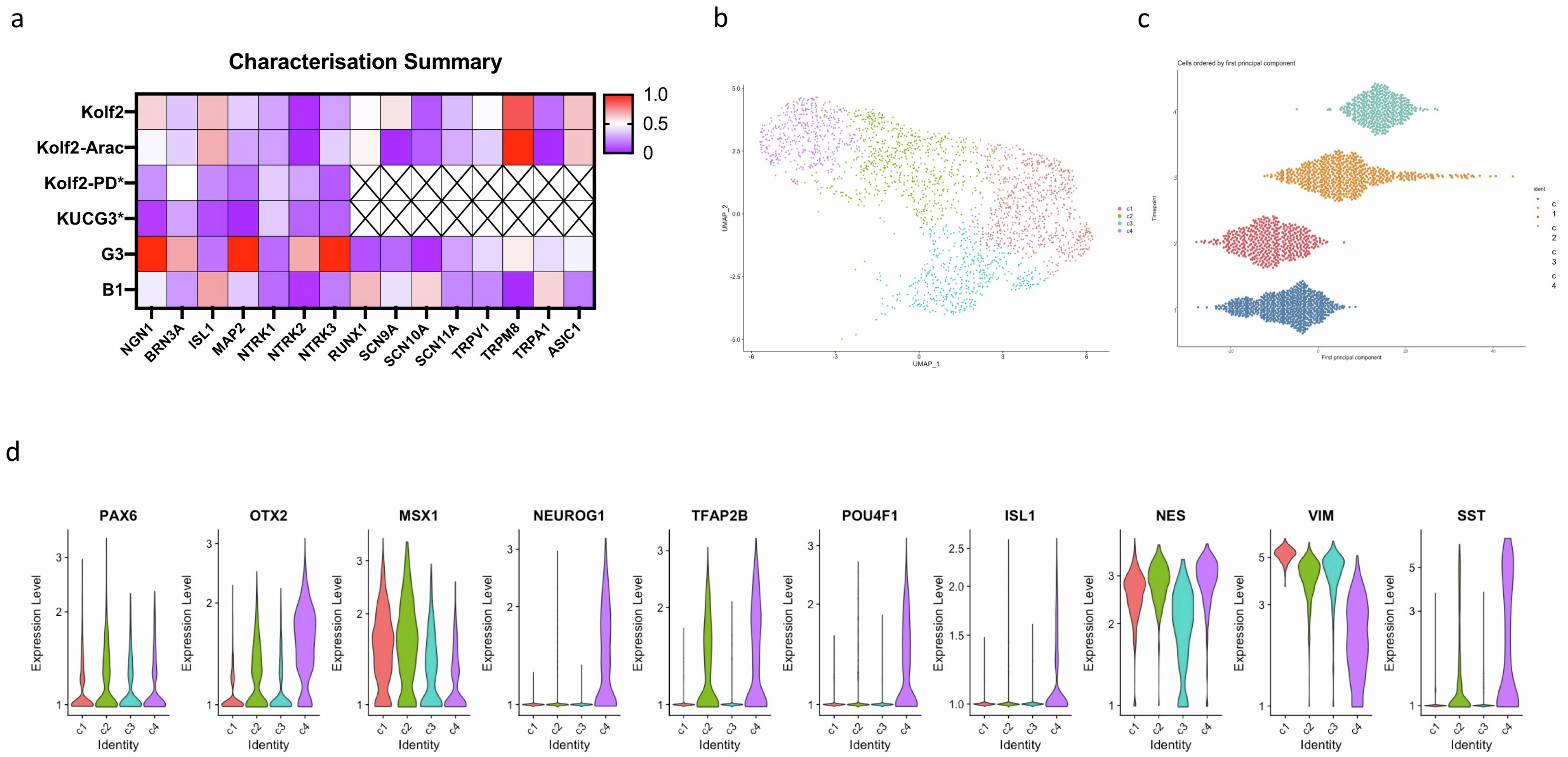

Figure S1 - Relating to figure 1. Single cell RNA sequencing of D11 sensory neuron precursors reveals the presence of distinct developmental states within the population. Preliminary experiments were performed on a range of iPSC lines to assess the viability of a previously described small molecule differentiation protocol. Data in **a** shows the normalised expression of a panel of key genes as determined by rt-qPCR. Data is from the final time point of the experiments (D35) except for those cell lines with a *, where data is from a precursor stage. Kolf2-Arac and Kolf2-PD represent differentiations where Arac-C and PD0332991 respectively were used as mitotic inhibitors. Unsupervised clustering of precursor cells using a nearest-neighbour based approach on the first 15 principle components of normalised gene counts revealed four clusters within the relatively homogenous body of cells. **(b)** shows UMAP dimension reduction plot of the sequenced cells and highlights the extent of each cluster. As the identified clusters represented groups of cells within the larger mass rather than distinct and separated groups, it was thought that the clusters may represent slightly different developmental stages of the same cell type. To confirm this, cells were assessed using a simple pseudo-time analysis based upon ordering of the first principle components (**c**). This revealed that c1 and c3 represent cells in an earlier developmental state, while cells in c2 and c4 appear as a more mature state. This was further confirmed with gene expression analyses presented in the main results. **(d)** shows violin plots of normalised gene expression, as determined by scRNA-seq, for a panel of key genes across the 4 identified clusters.

Figure S2

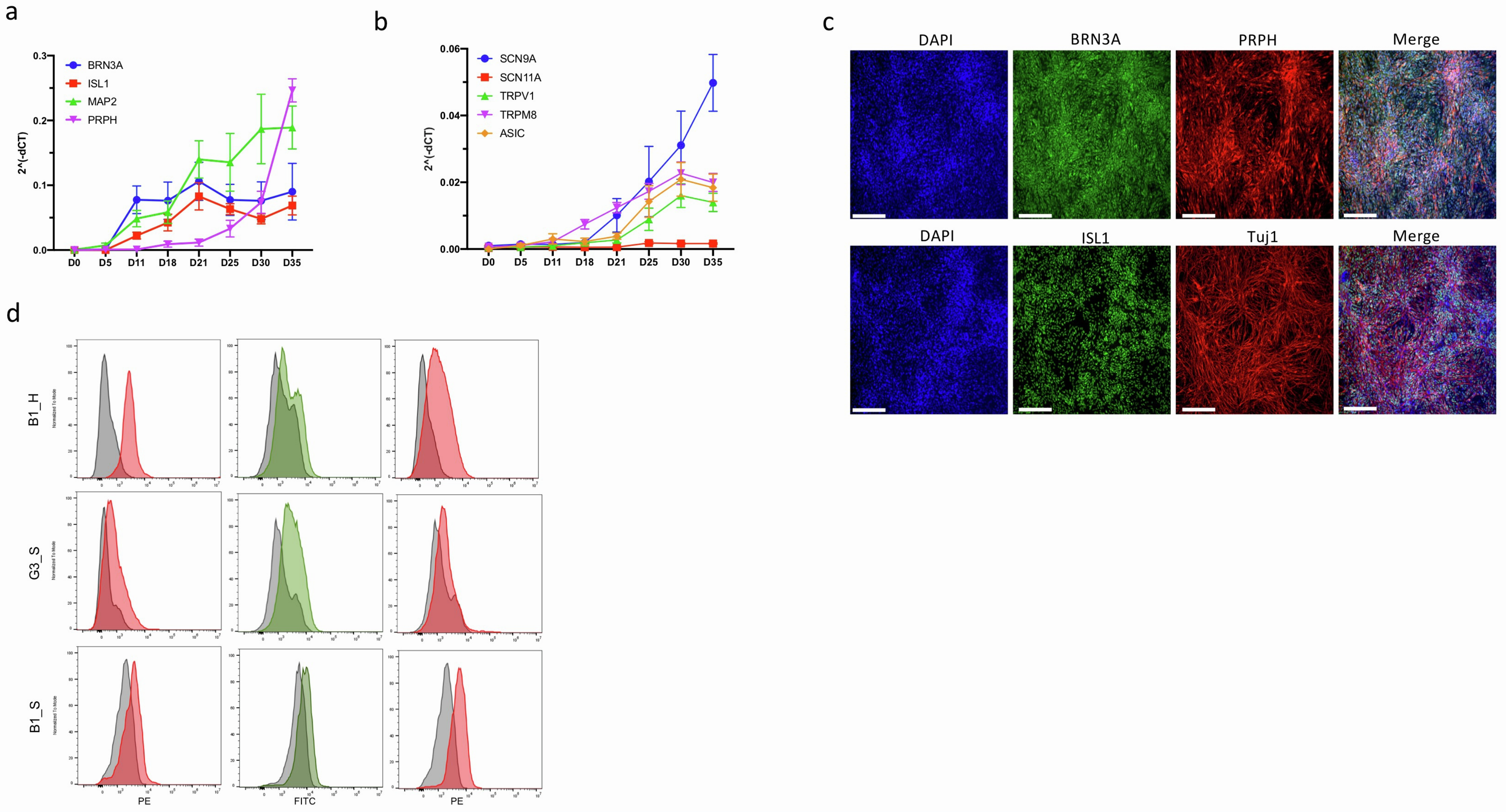

Figure S2 – Relating to figures 2 and 3. Key gene expression in sensory neurons differentiated from iPSCs using a hybrid protocol. **a&b** show developmental gene expression profiles with rt-qPCR from differentiations of G3 iPSC using our hybrid protocol. Data in both shows means ± SD. Panels in **c** show further representative immunohistochemistry images of G3_Hybird neurons at D35 showing expression of BRN3A, ISL1, PRPH and TUj1. Scale bars in all panels = 200 μm. Panel **d** shows representative histograms of cytometry data for the B1_H, G3_S and B1_S conditions. Data shows counts of cells based upon fluorescence intensity for each of the conjugated antibodies. Grey traces show unstained samples, coloured traces show stained samples. Counts are normalised to the mode for each.

Figure S3

a

Line	Protocol	Number of cells sequenced	Mean Reads/ cell	Mean features/cell	Percent mitochondrial genes
G3_H	Hybrid	4763	42503	3389	4.54
G3_S	Standard	3510	58744	4792	4.13
B1_H	Hybrid	5837	45793	3325	4.89
B1_S	Standard	7249	36846	2986	3.46

b

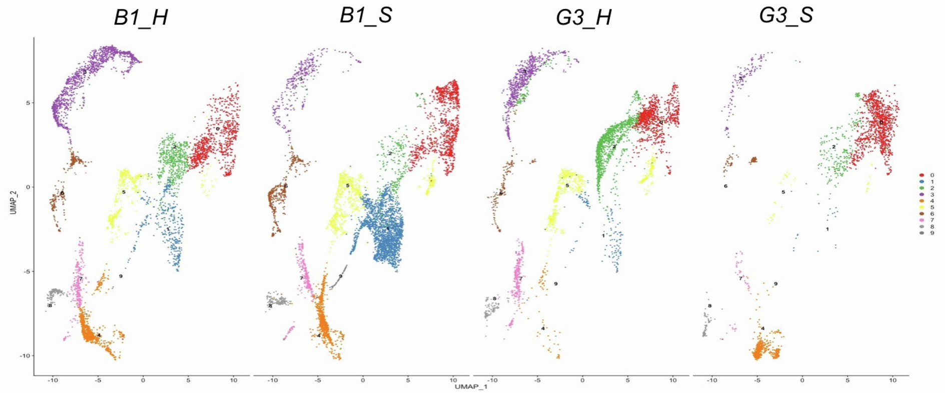

c

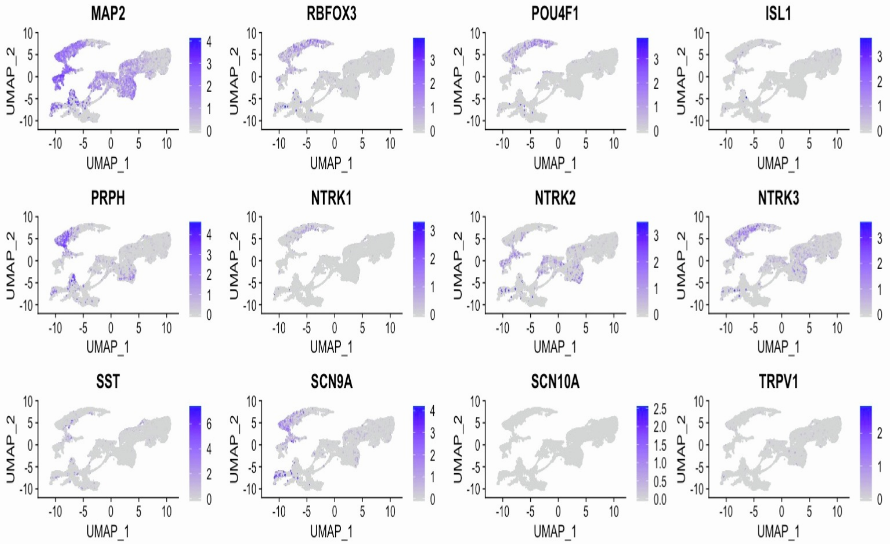

d

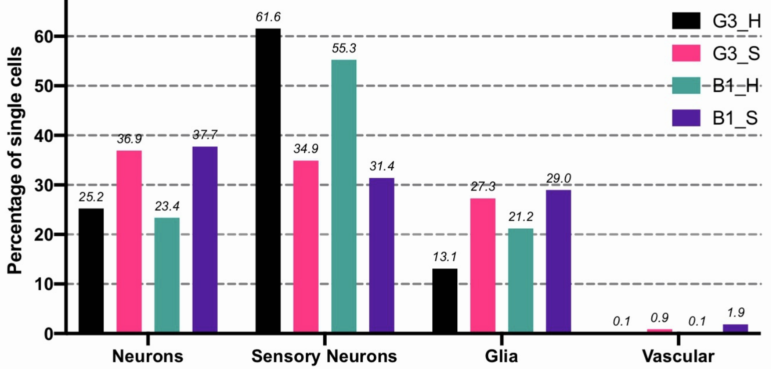

Figure S3 – Relating to figure 3. Additional characterisation of iPSC derived sensory neurons as determined by single cell RNA sequencing. Table in (**a**) describes the summary statistics relating to the single cell RNAseq libraires processed for each of the four conditions used in the study. Plots in **b** show UMAP dimension reduction plots of the full combined dataset, as in main Figure 3, where identified clusters are highlighted in colours and cells are split across panels based upon the original sample from which cells derived. **c** shows expression plots of key genes superimposed over UMAP dimension reduction plots, where each point represents one cell of the population. Colour gradient shows normalised expression. **d** presents the cell type assignment quantification results presented in main Figure 3e, grouped into more general cell-types to highlight differences between different cell lines and conditions.

Figure S4

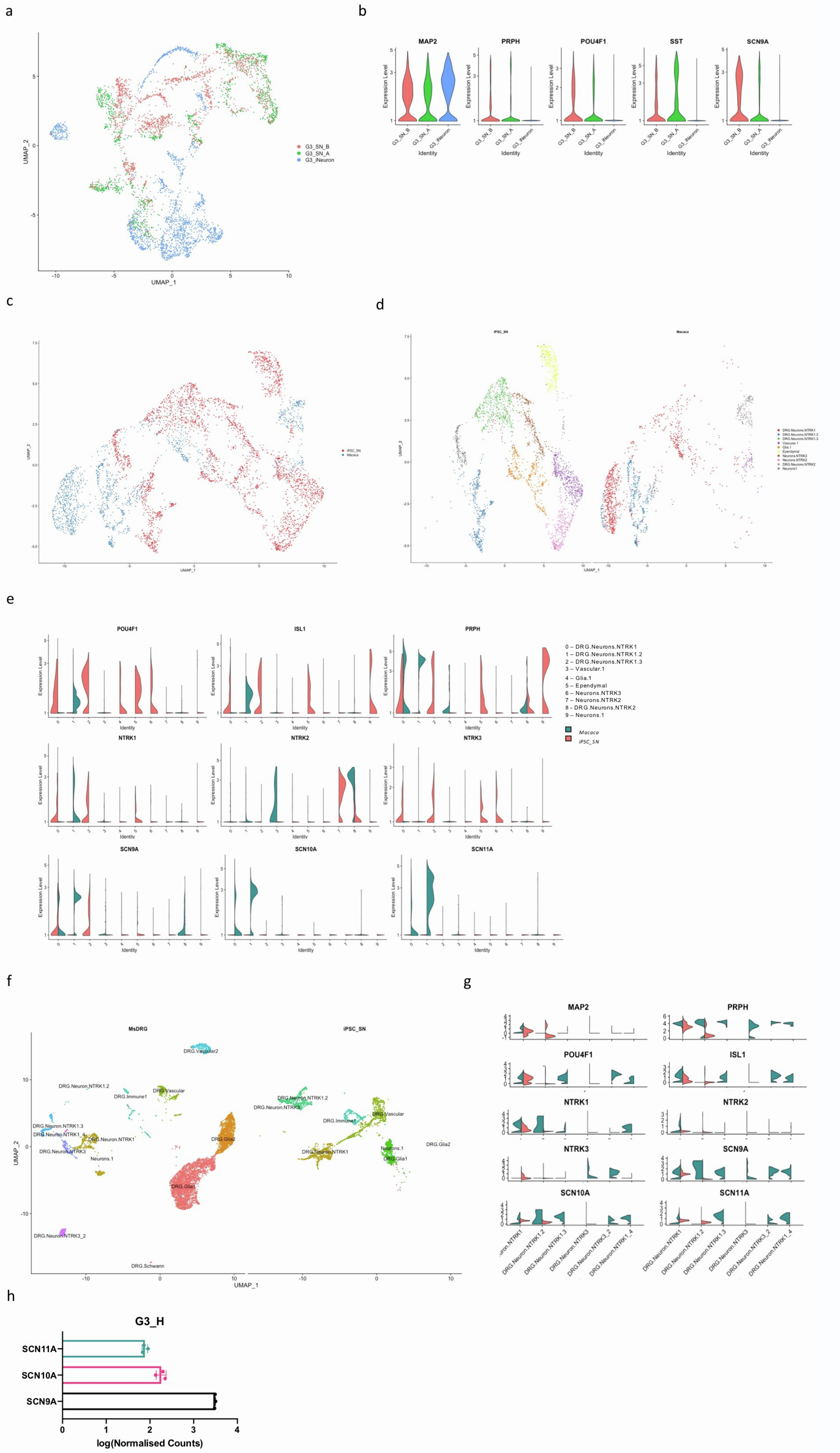

Figure S4 – Relating to Figure 3. Comparative single cell RNAseq analysis with alternative datasets. scRNA-seq datasets produced from iPSC derived sensory neurons were compared to two different data sets to allow further characterisation of cultures. A data set from G3 cultures using our hybrid protocol was first compared to cortical neurons from the same line, produced using an iNeuron protocol. Combined datasets were created by using an anchor-based integration approach. UMAP dimension reduction plots in **a** show the relationship of all cells in the combined dataset, grouped by the sample from which they were derived. Note, two individual sensory neuron datasets were used (G3_SN_A/B). Violin plots in **b** show the comparative expression of five key genes across the three individual datasets. To give an indication of the state of the differentiated cells compared to primary dorsal root ganglia (DRG), G3_Hybrid differentiated neurons were combined and clustered with a population of primary primate DRG cells. These were taken from a published dataset which used a SMRTseq2 platform. Datasets were processed, including normalisation and integration, as described in the main text. (**c&d**) present UMAP dimension reduction plots of the combined human/primate dataset, with **c** showing data split by species, and **d**, showing clusters identified using shared nearest neighbour-based clustering using the Louvain algorithm, parsed by the original sample. Cell populations were manually identified by analysis of the top differentially expressed genes for each cluster vs the remaining cells. **e** shows violin plots highlighting the expression of key genes across the clusters. G3_Hyrid differentiated neurons were also combined and clustered with a population of mouse DRG cells, processed for single cell RNA using the same 10X Genomics pipeline. (**f**) presents UMAP dimension reduction plot showing clusters identified using shared nearest neighbour-based clustering using the Louvain algorithm, split by the original sample source. Cell populations were manually identified by analysis of the top differentially expressed genes for each cluster vs the remaining cells. (**g**) shows violin plots highlighting the expression of key genes across the six identified clusters. (**h**) shows the log normalised counts of SCN genes from bulk RNA sequencing of G3 cells using the hybrid protocol. Samples were processed at D35. Plots show means +/- SD from three independent samples.

Figure S5

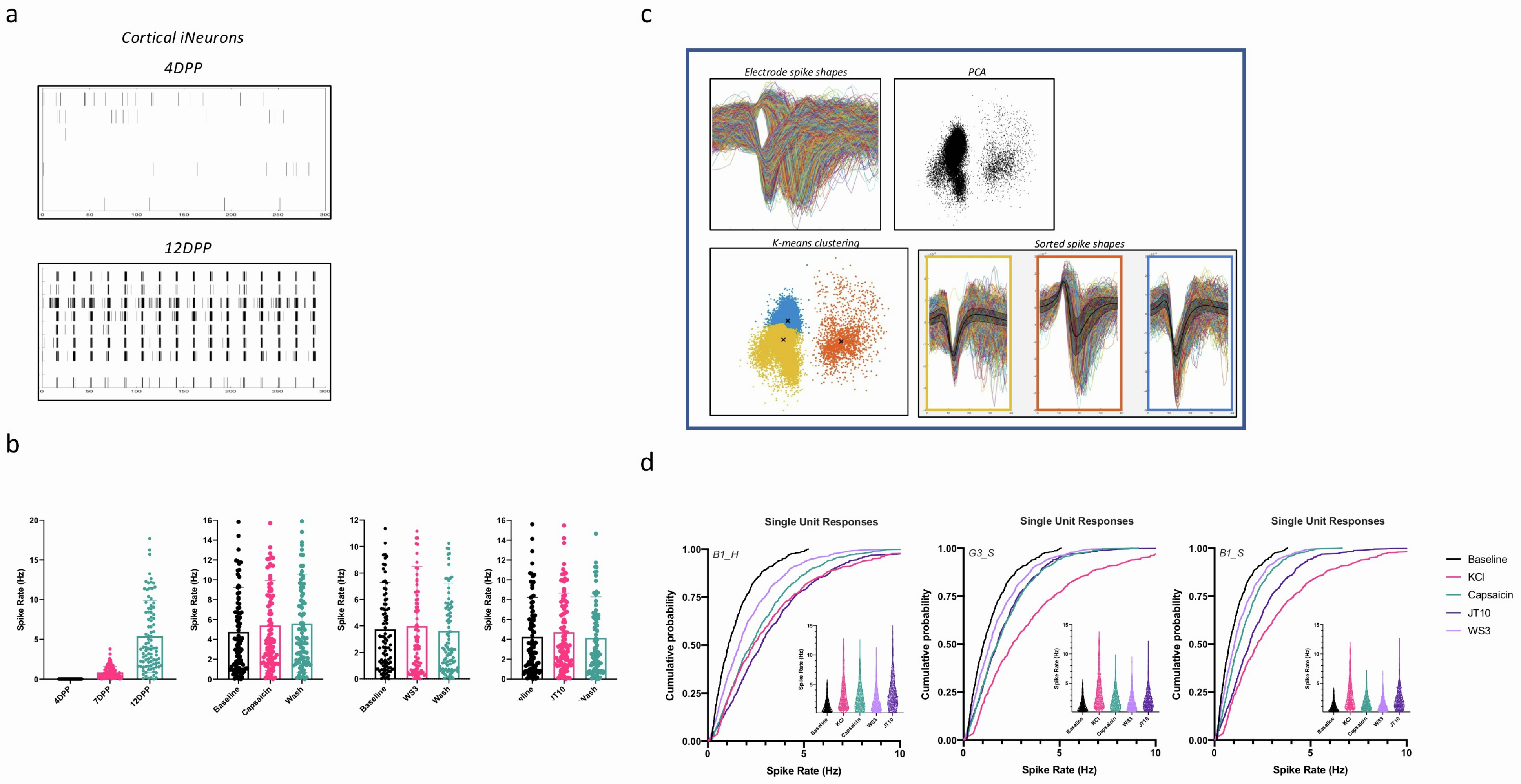

Figure S5 – Relating to figure4. Additional electrophysiology of stem cell derived sensory and cortical neurons (**a&b**) shows summary data of the spike rate of iPSC derived cortical neurons produced with an iNeuron protocol showing changes over development and when exposed to capsaicin, WS3 and JT10. Dots show the average spike rate of individual wells, plots show the median of each treatment group and error bars show SD. (**c**) Shows the steps involved in sorting spikes into multiple-unit components. While most electrodes record the activity of a single unit (single neuron), some detect activity from multiple cells, which often manifests in the appearance of multiple spike shapes. Dimension reduction using principle component analysis (PCA) is first performed on the amplitude of the spikes, the results of which are then used in a k-means unsupervised clustering algorithm. Cluster assignments are given to each waveform and the individual spikes are grouped accordingly to provide the true reflection of single unit activity in a well. Each group of waveforms and their associated statistics can then be considered a single unit. **d** shows single unit spike rate data from B1_H, G3_S and B1_S cultures. Main plots show cumulative probability of firing rates across the conditions, where KCl represents a QC treatment to identify all responsive cells; insets show violin plots of the responses where each dot shows the firing rate of a single neuron unit.

Figure S6

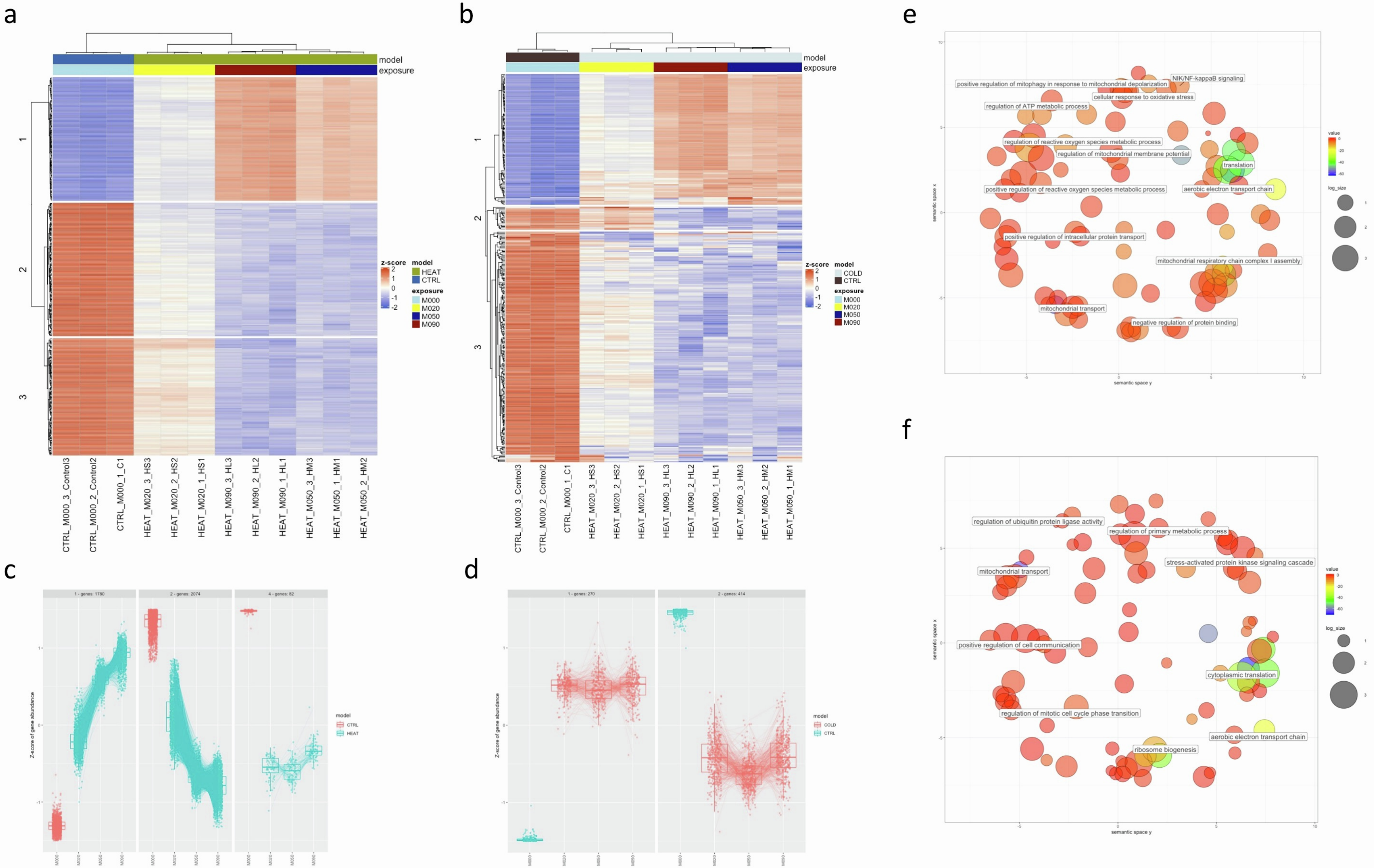

Figure S6 – Relating figure 5. RNAseq of stem cell derived sensory neurons exposed to temperature models of pain states. Plots in **a** and **b** show RNAseq results from experiments exposing neurons to 46°C and 5°C respectively, plotted as heat maps with samples clustered to exposure time. Data shows normalised and scaled data, presented as z-scores. **c** and **d** shows the results of top-down divisive hierarchical clustering of gene expression data form the heat and cold experiments respectively. Normalised gene expression is shown as zscores. Labels on x-axes represent the exposure time, where M000 = controls (before exposure), M020 = 20 mins, M050 = 50 mins, M90 = 90 mins and M600 = overnight exposure. Plots in (**e** and **f**) highlights the key enriched pathways as determined by analysis using Enrichr, followed by pathway condensation using Revigo, for heat and cold experiments respectively. Each bubble shows an enriched pathway, positioned according to its functional relationship to other GO terms, such that similar pathways are clustered together. The labels denote the representative pathways for each associated cluster. The size of each bubble shows the number of genes associated with each pathway term; the colour scale shows the normalized adjusted p-value of enrichment for each GO term.
